# Molecular Routes to Specific Identification of the Lactobacillus Casei Group at the Species, Subspecies and Strain Level

**DOI:** 10.3390/ijms21082694

**Published:** 2020-04-13

**Authors:** Piotr Jarocki, Elwira Komoń-Janczara, Agnieszka Glibowska, Michał Dworniczak, Monika Pytka, Agnieszka Korzeniowska-Kowal, Anna Wzorek, Monika Kordowska-Wiater

**Affiliations:** 1 Department of Biotechnology, Microbiology and Human Nutrition, University of Life Sciences in Lublin, 8 Skromna St., 20-704 Lublin, Poland; elwira.komon.janczara@up.lublin.pl (E.K.-J.); agnieszka.glibowska@up.lublin.pl (A.G.); m_dwor@tlen.pl (M.D.); monika.pytka@up.lublin.pl (M.P.); 2 Polish Collection of Microorganisms (PCM), Department of Immunology of Infectious Diseases, Hirszfeld Institute of Immunology and Experimental Therapy, Polish Academy of Sciences, Rudolfa Weigla 12, 53-114 Wroclaw, Poland; agnieszka.korzeniowska-kowal@hirszfeld.pl (A.K.-K.); anna.wzorek@hirszfeld.pl (A.W.)

**Keywords:** identification, differentiation, *L. casei* group, *L. rhamnosus*, *L. casei*, *L. paracasei*, probiotics

## Abstract

The genus *Lactobacillus* includes, among others, *Lactobacillus casei*, *Lactobacillus paracasei* and *Lactobacillus rhamnosus,* species that are collectively referred to as the *Lactobacillus casei* group. Many studies have shown that strains belonging to this group may decrease lactose intolerance, the effects of inflammatory bowel disease, diarrhea, constipation, food allergies and even colon cancer. Moreover, evidences exists of positive effects of these bacteria on mucosal immunity and blood cholesterol level. Because of their beneficial influence on human health, many of them are used as food additives and probiotic pharmaceuticals. It should be stressed that health-promoting properties are not attributed at the species level, but to specific strains. Therefore, procedures are necessary to allow specific identification at each phylogenetic level—genus, species and strain. In this paper we present a practical overview of molecular methods for the identification and differentiation of *L. casei* bacteria. The research included 30 bacterial strains belonging to three species: *L.casei,* *L. paracasei* and *L. rhamnosus.* Among the tested procedures were genus- and species-specific PCR, multiplex-PCR, Real-Time HRM analysis, RFLP-PCR, rep-PCR, RAPD-PCR, AFLP-PCR, and proteomic methods such as MALDI-TOF MS typing and SDS-PAGE fingerprinting. The obtained results showed that multiplex-PCR and MALDI-TOF MS turned out to be the most useful methods to identify the tested bacteria at the species level. At the strain level, the AFLP-PCR method showed the highest discriminatory power. We hope that the presented results will allow for the easy selection of an appropriate procedure, depending on the experiment conducted and the equipment capabilities of any given laboratory.

## 1. Introduction

The *Lactobacillus* genus belongs to a very large, heterogeneous group of lactic acid bacteria (LAB), that are commonly applied in the food and pharmaceutical industries and in agriculture [1,2]. The LAB includes, among others, *Lactobacillus casei*, *Lactobacillus paracasei* and *Lactobacillus rhamnosus* species, that are collectively referred to as the *Lactobacillus casei* group. These microorganisms are associated with habitats that are rich in nutrients such as fermented dairy products. They are also common inhabitants of the human gastrointestinal and urogenital tracts. Interestingly, some strains were also isolated from clinical patients with bacteremia, endocarditis, peritonitis and pneumonia [3,4].

Many studies show that strains belonging to *L. casei* group may induce physical health benefits in humans and livestock. It is commonly believed that these bacteria stabilize gut microflora, inhibit the development of pathogenic microorganisms, eliminate or minimize symptoms of lactose intolerance, prevent or alleviate the course of bacterial, viral and post-antibiotic diarrheas, as well as normalize disorders of gut peristalsis. Furthermore, it has been demonstrated that diet supplementation with probiotic bacteria may stimulate the human immune system and improve regulation of blood cholesterol [3,5,6]. Owing to the imposed ban on the use of antibiotic growth stimulants in animal feed, increasing importance is ascribed to studies of other additives permitted for use in feed. The use of probiotics in animal feed improves the effectiveness of agricultural production, as it results in the increased digestibility of feedstuffs and synthesis of some vitamins, thereby increasing body weight gain, as well as improving the health of animals, increasing their resistance to stress conditions, and ensuring faster recovery from disease [7,8].

The isolation of novel strains, and ascertaining their characteristics in terms of health-promoting traits, allows the introduction of innovative, competitive probiotic preparations to the market, with ever increasing applications. During strain isolation, it is crucial to consider the problem of potential repeat isolation of the same strain. This limitation may significantly affect the cost-effectiveness and labor-consumption of the isolation and selection of new bacterial strains. What is more, the production of food containing additives in the form of live microorganisms requires exceptional control, allowing the rapid detection of possible contamination. These negative occurrences may be minimized through the use of methods that allow for rapid identification and differentiation of isolates.

In this regard, methods that enable discrimination of bacteria at the strain level are of particular interest [9,10,11]. Considering that tens, or even hundreds of strains isolated from the same source are usually subjected to differentiating analyses, the choice of differentiating technique is typically driven by factors such as the duration and cost of the analysis. Today, commonly applied methods include those based on phenotypic analysis derived from genetic expression (e.g., analysis of protein profiles with SDS-PAGE or MALDI-TOF MS) as well as methods based on DNA analysis, e.g., restriction analysis of chromosomal DNA with pulsed-field electrophoresis—PFGE, restriction analysis of amplified selected genome regions—RFLP, or methods based on the amplification of repetitive sequences (Rep-PCR) or random sequences (RAPD-PCR). These techniques differ in both their differentiating potential and time requirements (duration). The price of equipment necessary to conduct these tests and the cost of the analysis itself is also noteworthy [11,12,13].

Numerous studies have shown the effectiveness of selected molecular methods in identifying bacteria belonging to the *L. casei* group [14]. Nevertheless, these studies were carried out using a range of different bacterial strains, and used very different procedures for identification and differentiation. Consequently, when analyzing the extensive scientific literature, it is difficult to unequivocally assess the effectiveness of individual methods in genotyping *L. casei* group strains. In this paper, we present a comparison of the most popular molecular techniques, which are not based on direct analysis of single gene sequences or whole genomes. Thirty strains of bacteria that were originally classified into three species, *L. casei*, *L. paracasei* and *L. rhamnosus,* were used in the study. We hope that the presented results will help to organize the knowledge on identification and differentiation of *L. casei* group microorganisms, and will simplify the choice of identification procedure applicable to the characteristics of future research being conducted with the use of these extremely important industrial bacteria.

## 2. Results and Discussion

### 2.1. Genus, Species and Subspecies-Specific PCR

In the first stage of this study, we tested the usefulness of previously developed methods based on PCR, using generic primers (specific at the genus level yet not at the species level). To confirm the affiliation of the test strains to the genus *Lactobacillus*, two sets of primers designed based on the 16S-23S spacer region rDNA sequence [15], and the *tuf* gene, which encodes elongation factor Tu [16] (Table S1), were used. As expected, target PCR products of about 250 bp and 800 bp were obtained respectively for all tested strains (Table 1). The obtained results clearly confirmed that the test isolates belong to the genus *Lactobacillus*.

In the subsequent test, *L. casei* specific primers (Table S1) [17] were used in the PCR reaction. Such an experimental system seems to be particularly useful when isolating new bacterial strains, and when it is necessary to quickly and specifically confirm that they belong to the *L. casei* group. Interestingly, in this test, a specific amplicon of about 364 bp was obtained for 29 of the thirty tested strains of bacteria (Table 1). Despite several attempts, no positive result was obtained for strain JCM 8677, which may suggest that the isolate was misclassified as *L. casei*.

These assumptions were confirmed in the next stage, which consisted of identifying individual species (*L. casei*, *L. paracasei* and *L. rhamnosus*) belonging to the L. casei group [18,19]. For this purpose, a set of 5 primers was used, which consisted of one universal and 4 species-specific oligonucleotides, one of which was designed for the *L. zeae* taxon, which has since been reclassified within *L. casei* [20]. The primers are all based on the 16S rRNA, which is the primary region used to identify microorganisms through PCR. The expected specific PCR product was fewer than 300 nucleotides (290 bp) long (Table S1). From the beginning of the analysis, we were surprised that only 6 out of 10 strains originally classified as *L. casei* obtained a positive result in the form of a specific PCR product (Y2 and casei primers) (Table 1). For the remaining isolates and strains belonging to *L. paracasei* and *L. rhamnosus*, either no amplicon or numerous non-specific products were obtained. a positive result in the form of a specific PCR product (Y2 and casei primers) (Table 1). For the remaining isolates and strains belonging to *L. paracasei* and *L. rhamnosus*, either no amplicon or numerous non-specific products were obtained.

A second set of primers (Y2, para) designed to identify the species *L. paracasei,* made it possible to confirm that all strains that were originally classified as this species did belong to it. Additionally, 2 out of 10 putative *L. casei* strains (JCM 2120 and JCM 20024) also produced a positive result. Interestingly, these were the strains for which no specific amplicon with Y and cas primers was obtained. Additionally, in the case of the PCR reaction specific to *L. rhamnosus* (Y2 and rham primers), further to the 10 strains which belonged to this species, a positive result was also obtained for strain JCM 8608 (identified as *L. casei*). The remaining microorganisms belonging to *L. casei* and *L. paracasei* did not generate specific products of expected length in this test.

The final set of oligonucleotides (Y2 and zeae primers) was designed to quickly identify isolates belonging to “*L. zeae*”. In our study, out of 30 strains, only *L. casei* LMG 17315 was included in the taxon in question on the basis of previous reports [20,21]. Interestingly, the results showed that the experimental system being tested generated a specific PCR product for all *L. casei* strains whose species had been confirmed in reactions with Y2 and cas primers. The obtained results suggest that the sole use of Y2 and zeae primers (D1) does not enable *L. casei* strains to be distinguished from bacteria belonging to “*L. zeae*”.

Huang et al. [22], on the basis of RAPD profiles, developed primers that generated positive results only for “*L. zeae*” strains. Using a similar reaction system in our research, we also only obtained positive results for strain LMG 17315. In the same study, Huang et al. also developed specific primers for *L. rhamnosus* and *L. paracasei subsp. tolerans*. By testing these primers in our study, similarly to the Y2 and rham primers, we were able to confirm the phylogenetic relevance of eleven strains (*L. rhamnosus*) (Table 1). After numerous tests on 1 of the 2 strains of *L. paracasei* and *tolerans* sub-species, a positive result was obtained in reaction with primers specific to this subspecies (SpeOPT11tol-F and SpeOPT11tol-R) (Table 1).

In summary, a complex of primers based on the 16S rRNA region made it possible to confirm species affiliation in 26 of the 30 microorganisms tested. For strain JCM 8677, no positive results were obtained in any of the tests, which confirms previous results suggesting that it is not a member of the *L. casei* group. Strains JCM 2120, JCM 20024 and JCM 8608, which were originally proposed as *L. casei* species (based on 16S rRNA gene sequence), probably belong to other species within the *L. casei* group, namely *L. paracasei* and *L. rhamnosus* respectively (Table 1). A similar problem with the classification of *L. casei* group was recently described in detail by Wuyts et al. [5].

The identification of bacteria based on ribosomal DNA appears to be a very useful tool for identifying the bacterial species belonging to the *L. casei* group. Nevertheless, due to the genetic similarity of the three species, in many cases inconclusive results have been obtained in the form of a mixture of numerous non-specific products and specific products of low intensity, which may contribute to misidentification. To avoid incorrect identification, experimental systems based on a sequence of differentiated genes, such as *dnaK* and *tuf*, may be used [23,24,25]. Methods such as RFLP-PCR, Multiplex PCR or HRM-PCR, which are predominantly based on the simultaneous identification of species in one reaction, also seem to be good alternatives. These techniques were tested in the next stage of the research.

### 2.2. Rapid Distinguishing of Three Species Belonging to the L. casei Group Using RFLP-PCR, Multiplex PCR and HRM-PCR

#### 2.2.1. Restriction Fragment Length Polymorphism

Earlier results have shown that, due to the high similarity of *L. casei*, *L. paracasei* and *L. rhamnosus*, quick and unambiguous identification of individual species can be quite challenging. Genotypic methods, such as restriction fragment length polymorphism (RFLP) [26], have been used for this purpose for years. The advantage of this technique is its great simplicity and lack of a need for sophisticated apparatus [27]. In the first stage, the selected gene is amplified, then the obtained products are digested with a specific restriction enzyme, ending with electrophoretic analysis of the products obtained as a result of the effect of restrictases. When the amplified gene is from the 16S rRNA, this method is often called amplified ribosomal DNA restriction analysis (16S-ARDRA) [13].

In this work, the 16S rRNA of 30 bacterial strains was amplified, following which the obtained PCR reaction products of approximately 1500 bp were treated with the enzyme MseI (5’-T^TAA-3’). Such a system was used, among others, in the work of Duskova et al. [28] in which the authors successfully identified lactobacilli isolated from food. Analysis of the results obtained showed that this procedure had insufficient differentiation strength to unequivocally identify the test species. This is particularly evident in Figure S1A, in which the DNA profiles obtained from 10 strains originally classified as *L. casei* species can be observed. As earlier results have shown, 2 of these strains belonged to *L. paracasei* (JCM 2120 and JCM 20024) and one to *L. rhamnosus* (JCM 8608). For all these strains, identical or very similar profiles were obtained to the results obtained for *L. casei* strains. The only microorganism that stood out in this test was JCM 8677 which, as previously stated, does not belong to the *L. casei* group.

The results obtained for *L. rhamnosus* strains show that, in some cases, the ARDRA method can even generate DNA profiles characteristic of individual strains. This phenomenon may be caused by point mutations in the amplified gene. In the case of ARDRA, the probability of such a polymorphism is high, because the amplified target region (within the 16S rRNA) usually appears in several copies in the bacterial genome [26]. In short, the procedure we tested (digestion of a 16S rRNA amplicon with MseI) proved to be ineffective for quick identification of *L. casei* species. Dec et al. [27], showed that digestion of 16S rRNA amplicons with MseI and HinfI enzymes could be more effective for this purpose. In this paper, however, we have chosen another solution in which we subjected *tuf* and *dnaK* genes to restriction digestion, whose sequences show greater variability among the examined species compared to the 16S rRNA ribosomal gene.

**Figure 1:**
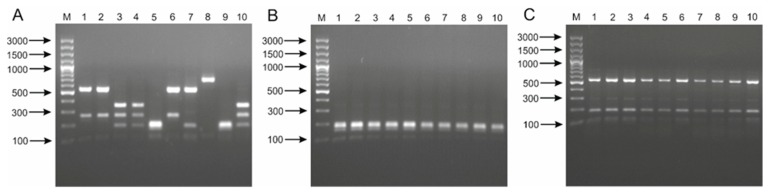
Restriction fragment length polymorphism (RFLP) patterns generated from restriction analysis of *tuf* amplicon (~800 bp) of 30 strains belong to *L. casei* group using HaeIII restrictase. Analysis of the discriminatory power of the procedure applied was performed for 10 strains of *L. casei* (**A**)—M, DNA molecular marker; 1, LMG 6904; 2, LMG 23516; 3, LMG 24099; 4, LMG 24102; 5, JCM 2120; 6, JCM 8129; 7, JCM 8608; 8, JCM 8677; 9, JCM 20024; 10, LMG 17315; 10 strains of *L. paracasei* (**B**)—M, DNA molecular marker; 1, LMG 13087; 2, LMG 9193; 3, LMG 9438; 4, LMG 11459; 5, LMG 11961; 6, LMG 12164; 7, LMG 19719; 8, JCM 1163; 9, LMG 9191; 10, JCM 20315; and 10 strains of *L. rhamnosus* (**C**)—M, DNA molecular marker; 1, LMG 6400; 2, LMG 8153; 3, LMG 10768; 4, LMG 10772; 5, LMG 12166; 6, LMG 18030; 7*,* LMG 23304; 8, LMG 23536; 9, LMG 23550; 10, LMG 25881.

In the case of the PCR products obtained for the *tuf* gene (800 bp), an enzyme recognizing the four-nucleotide sequence 5’-GG^CC-3’—HaeIII was used for restriction digestion [16,29]. Although, in comparison to ARDRA, a smaller number of bands was obtained, different DNA profiles were obtained for all the examined species (Figure 1). Interestingly, for the *L. casei* strains, two different sets of digest products were obtained (Figure 1A; Lines 1, 2, 3, 4, 6 and 10), which indicates intra-species variation in *tuf* gene sequences. However, this phenomenon may hinder clear identification of the species. In the final experimental system tested, the *dnaK* gene, encoding the 70-kDa heat shock protein (HSP70), which is considered a very useful genetic marker in bacterial phylogenetics studies, was replicated [23,30]. A bioinformatics analysis showed an average variation of 87.8% in *dnaK* sequences among type strains of species belonging to the *L.casei* group, which, compared to 99.1% variation in the 16S rRNA genes, seems to predispose this gene for use in the RFLP procedure [29]. The obtained amplicons, each approximately 995 bp in length, were digested with the ApoI enzyme (5’-R^AATTY-3’), and the digest products were separated in agarose gel. As in the study by Park et al. [29], characteristic species-specific restriction profiles were observed for *L. casei, L. paracasei* and *L. rhamnosus* species. The results obtained were characterized by high reproducibility, confirmed by DNA profiles obtained for all 30 strains (Figure S2). Interestingly, a slight but noticeable difference was observed for strain LMG 17315, which was previously classified as “*L. zeae*,” and five other isolates designated as *L. casei* (Figure S2A). On the other hand, it was not possible to obtain results which would enable the differentiation of two sub-species of *L. paracasei* (Figure S2B). The observations made confirm earlier reports that RFLP is an effective and very reproducible method that enables identification of bacteria, mainly at the species level [13,29].

#### 2.2.2. Multiplex PCR

One alternative method to PCR-RFLP, that allows for rapid identification of different, even closely related species of microorganisms is multiplex PCR. The advantage of this method is its simplicity and the absence of additional stages, such as restriction digestion. On the other hand, especially for bacteria with high genetic similarity, the design and optimization of the reaction itself can be relatively difficult [31,32]. In this study, we used two sets of primers that were designed based on the *tuf* and *mutL* gene sequences (Table S1), to identify species of the *L. casei* group [16,33].

In the first experimental system, we replicated methods by Ventura et al. [16], who obtained species-specific profiles of PCR products; three products for *L. casei* (700, 540 and 350 bp), two for *L. paracasei* (540 and 240 bp) and a single amplicon for *L. rhamnosus* (540 bp). This same procedure was used in a species-by-species analysis of 30 tested strains. For 29 test subjects, it was possible to clearly determine their identification as one of 3 species—*L. casei*, *L. paracasei* and *L. rhamnosus*. Interestingly, and similar to findings of a study by Iacumin et al. [34] for strains belonging to *L. casei*, a slightly different length spectrum of PCR products (350, 520, 600 and 1000 bp) was obtained. Despite this, the observed electrophoretic profiles were still characteristic of the species (*L. casei*). In the case of strain JCM 8677, whose membership of the *L. casei* group has not been confirmed, no positive results were obtained. For the remaining analyzed strains belonging to *L. paracasei* and *L. rhamnosus,* a characteristic banding pattern was obtained, allowing for unambiguous confirmation of the original species classification.

The second set of primers described by Bottari et al. [33] made it possible to generate single, species-specific PCR products of 666 bp (*L. casei*), 253 bp *(L. paracasei*) and 801 bp (*L. rhamnosus*). This multiplex PCR reaction confirmed the taxonomic relevance of 28 tested strains. As in the previous reaction, no specific PCR product was obtained for JCM 8677, and for JCM 8608, which as shown in previous experiments probably belongs to *L. rhamnosus*. Preliminary tests carried out in our laboratory showed that the procedure described by Bottari et al. may lead to false negative results in some cases (e.g., for *L. rhamnosus* strains). Of the 29 strains belonging to *L. rhamnosus*, PCR products of the expected length could not be obtained in six cases (data not shown). Similar cases are also described by Bottari et al. [33].

#### 2.2.3. High-Resolution Melting Analysis

The final method tested in this study to quickly differentiate strains of the *L. casei* group into different species was HRMA. Because there is no need for electrophoretic separation, the great advantage of this method is its speed and single-step execution, which in cases where there are a large number of new isolates targeted for identification, significantly reduces the possibility of potential confusion [35,36]. On the other hand, the weakness of the technique lies in the fact that monitoring the melting temperature of PCR products requires special equipment and software. However, it appears that equipment for performing qPCR with the possibility of HRM analysis has now become basic equipment for many labs, so the use of this method for standard bacterial genotyping is becoming increasingly available [37,38].

**Figure 2:**
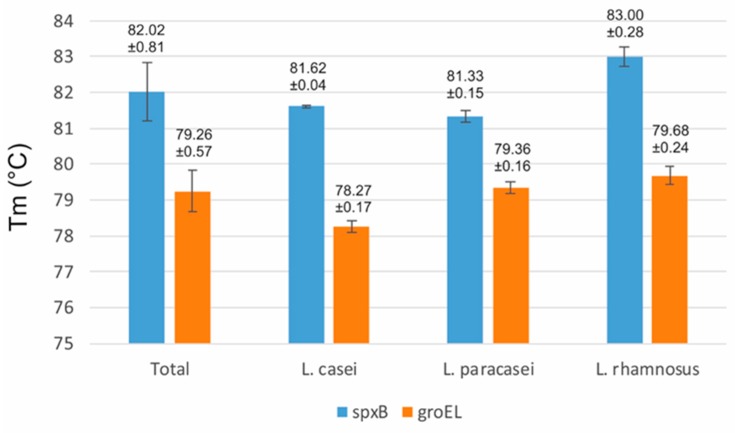
Average melting temperature of *spxB* and *groEL* amplicons obtained for 30 bacterial strains belong to *L. casei*, *L. paracasei* and *L. rhamnosus* species.

In this study, two sets of primers, which were developed based on *spxB* and *groEL* gene sequences, were used to differentiate three closely related species [39,40]. For the first experimental system (*spxB)* the following average melting temperatures were obtained: 81.6 ± 0.04 °C for *L. casei*, 81.3 ± 0.15 °C for *L. paracasei* and 83 ± 0.28 °C for strains belonging to *L. rhamnosus* (Figure 2). Detailed results for individual strains are shown in Table 1. The analysis showed that the method used allowed unambiguous identification of strains belonging to the species *L. rhamnosus*. For the isolates belonging to *L. casei* and *L. paracasei,* the obtained melting temperatures of PCR products were similar. For 3 *L. paracasei* strains (LMG 11961, LMG 19719 and JCM 1163), the result was 81.5 °C and for *L. paracasei* LMG 13087 the result was 81.6 °C (Table 1). Obtaining such results for new isolates could lead to their incorrect classification. It should also be noted that our mean Tm values for individual species differed significantly from the analogous results obtained by Sardaro et al. [40]. Such an observation puts a big question mark over the possibility of comparing results obtained from different laboratories using the described procedure.

The second set of primers made it possible to amplify a fragment of the *groEL* gene, of about 150 bp in length. In earlier studies, Koirala et al. [39] obtained a mean Tm for *L. casei* of 78.22 ± 0.15 °C, 79.10 ± 0.27 °C for *L. paracasei* and 79.60 ± 0.17 °C for *L. rhamnosus*. Interestingly, the mean Tm values for the species studied in our study were very similar to the above, and were in turn: 78.27 ± 0.17 °C (*L. casei*), 79.36 ± 0.16 °C (*L. paracasei*) and 79.68 ± 0.24 °C (*L. rhamnosus*) (Figure 2). Furthermore, analysis of the detailed results showed that, based on the melting temperatures obtained for the PCR products, the classification of 27 of the 29 bacterial strains belonging to the group *L. casei* could be carried out quickly and clearly (Table 1). Only for two *L. rhamnosus* strains (LMG 18030 and LMG 23536) was a lower Tm value obtained; 79.0 °C and 79.5 °C, respectively, which suggested that they belonged to *L. paracasei*. It should also be noted that in both tests no specific PCR products were obtained for strain JCM 8677, which is consistent with previous results.

Analyzing the results obtained, it can be concluded that both procedures are very useful for the identification of *L. casei* species. Only for a small number of strains would a confirmation procedure be necessary for accurate identification. Interestingly, through collective analysis of detailed results from both experiments, it was possible to identify all tested strains belonging to the *L. casei* group. Such results suggest that a very effective solution would be to use both tested methodologies simultaneously.

Summarizing this stage of research, on the basis of the results obtained, it can be concluded that the most useful technique for rapid differentiation of *L. casei*, *L. paracasei* and *L. rhamnosus* species seems to be multiplex PCR. On the other hand, in rare cases this method generated false negative results, e.g., for some *L. rhamnosus* strains, which required an additional confirmation step. HRM-PCR also seems to be a very quick and thus convenient tool. However, when identifying taxa that are very closely related, it should be expected that for some strains uncertain results may be obtained. Based on the results obtained using the PCR-RFLP technique, it can be concluded that not all of the experimental systems used made it possible to identify the tested bacteria at the species level. The system in which PCR products obtained by *dnaK* gene amplification were digested with the restriction enzyme ApoI proved to be the most effective. On the other hand, due to the introduction of restriction digestion, which extends the analysis time, this method seems to be a good alternative as a confirmatory tool in cases with ambiguous results from other analyses, such as multiplex PCR or HRM-PCR.

### 2.3. Fingerprinting Typing—RAPD, Rep-PCR, AFLP

#### 2.3.1. Random Amplification of Polymorphic DNA

As mentioned previously, research to confirm the health-promoting properties of strains used as probiotics requires effective and precise methods to identify microorganisms at the strain level. Furthermore, during the isolation of novel microorganisms from the same source, techniques that allow identical isolates resulting from multiple isolation of the same strains to be quickly excluded from the research process seem to be important. Therefore, it is very important to use methods that combine both a high ability to differentiate and simplicity of execution, so that large numbers of isolates can be analyzed simultaneously. The time and financial costs of such analyses are also important [41,42].

The first method used in this study, which was tested to differentiate the tested bacteria at the strain level, was the random amplification of polymorphic DNA—RAPD. It is a very simple, even primitive method, using short oligonucleotides to amplify many DNA fragments, which after electrophoretic separation should represent a profile of PCR products characteristic of the microorganism. Numerous studies have shown the effectiveness of this method in differentiating closely related microorganisms [13,43]. Moreover, the obtained DNA profiles have in many cases been used to develop primers to identify bacteria at the species and even strain level [22,44]. On the other hand, it should be stressed that the disadvantage of this type of analysis is the relatively low reproducibility, which may result from even slight changes in the composition of the reaction mixture or temperature profile of the PCR reaction [45].

**Figure 3:**
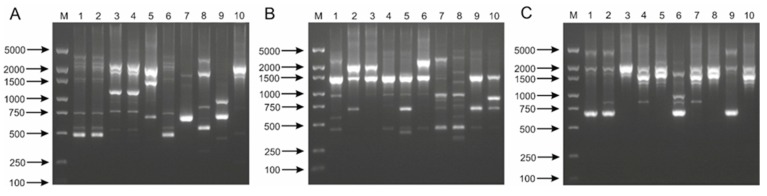
Randomly amplified polymorphic DNA (RAPD)-PCR patterns obtained with 80B_RAPD_M13 primer for 30 lactobacilli belong to *L. casei* group. Analysis of the discriminatory power of the procedure applied was performed for 10 strains of *L. casei* (**A**)—M, DNA molecular marker; 1, LMG 6904; 2, LMG 23516; 3, LMG 24099; 4, LMG 24102; 5, JCM 2120; 6, JCM 8129; 7, JCM 8608; 8, JCM 8677; 9, JCM 20024; 10, LMG 17315; 10 strains of *L. paracasei* (**B**)—M, DNA molecular marker; 1, LMG 13087; 2, LMG 9193; 3, LMG 9438; 4, LMG 11459; 5, LMG 11961; 6, LMG 12164; 7, LMG 19719; 8, JCM 1163; 9, LMG 9191; 10, JCM 20315; and 10 strains of *L. rhamnosus* (**C**)—M, DNA molecular marker; 1, LMG 6400; 2, LMG 8153; 3, LMG 10768; 4, LMG 10772; 5, LMG 12166; 6, LMG 18030; 7*,* LMG 23304; 8, LMG 23536; 9, LMG 23550; 10, LMG 25881.

In this study, four experimental systems that have been presented in previous studies were used to differentiate *L. casei* group strains (Table S1). On the basis of the results obtained, for 6 strains belonging to *L. casei*, it can be concluded that the method used is not effective enough to differentiate the strains of this species. In all experimental systems (four primers), 3 different DNA profiles were obtained for the tested strains (Figure 3 and Figures S3–S5). Identical profiles were obtained for strains LMG 6904, LMG 23516 and LMG 8129 as well as LMG 24099 and LMG 24102. Characteristic profiles were obtained only for strain LMG 17315, which in some works is described as a separate taxon “*L. zeae*”. It is worth stressing here the high reproducibility of the obtained results, regardless of the applied reaction system.

For *L. paracasei* strains, the best results were obtained with M13 primer [46], which produced characteristic DNA profiles for all tested microorganisms (Figure 3B). Other experimental systems were characterized by lower differential strength; for some strains smaller amounts of PCR products were obtained or PCR products of similar length and low intensity were observed (Figures S3–S5). Referring to the results obtained for two strains belonging to *L. paracasei* subsp. *tolerans* subspecies, it should be observed that similar DNA profiles were obtained in each reaction system, however, using primers 80B_RAPD_M13 and 80C_RAPD_OPT-14 it was possible to clearly differentiate them (Figure 3B and Figure S4B). It should also be noted that in the case of the 80A_RAPD primer, a PCR product with a length of about 650 bp was obtained, which was characteristic only for the *L. paracasei* subsp. *tolerans* (Figure S3B). On the basis of such results, it is possible to design primers specific to the above subspecies.

Using the 80A_RAPD primer and DNA samples isolated from *L. rhamnosus* strains, similar DNA profiles were obtained to those obtained with 80B_RAPD_M13 and 80C_RAPD_OPT-14. However, in some cases slight differences between strains could be observed (Figure S3C). For three isolates, LMG 10772, LMG 23304 and LMG 25881, the observed PCR products had exactly the same lengths. Also, strains LMG 6400 and LMG 23550, as well as LMG 10768 and LMG 12166 showed great similarity in this test. Interestingly, for all tested *L. rhamnosus* bacteria a characteristic PCR product of just over 1000 bp in length was obtained. Comparing the results obtained in this reaction with the 80A_RAPD and 80B_RAPD_M13 primers, we observed that in the second case it was possible to distinguish strain LMG 10768 from the LMG 12166 isolate, as well as strain LMG 25881 from LMG 10772 and LMG 23304. On the other hand, for strains LMG 6400 and LMG 23550, as well as LMG 10772 and LMG 23304, identical DNA profiles were again obtained (Figure 3C). Similar results were also observed in reactions with the 80C_RAPD_OPT-14 primer (Figure S4C). The final primer, 80_D_RAPD_OPA-18, generated very similar PCR products for all tested strains under applied reaction conditions. It is worth noting that, in each case, a species-specific product of ~1000 bp was obtained (Figure S5C).

In conclusion, the results obtained confirm the high potential of RAPD-PCR in bacterial differentiation at the strain level. The highest differential strength was characterized for a system in which 80B_RAPD_M13 primer was used. In this case, it was possible to reproducibly differentiate all of the strains belonging to *L. paracasei* and most of the strains belonging to *L. casei* and *L. rhamnosus*. Other reaction systems were characterized by lower differentiation potentials, which may have been influenced by parameters such as primer choice and polymerase concentration or the applied PCR temperature profile. The surprisingly high reproducibility of the results should also be stressed. In individual RAPD-PCR tests for strains which were characterized by high similarity, identical or very similar electrophoretic profiles were usually obtained. As the literature has shown, in order to differentiate similar strains, two or more primer pairs can be applied in one reaction, which may increase the differential strength of the method used [47].

#### 2.3.2. Rep–PCR

**Figure 4:**
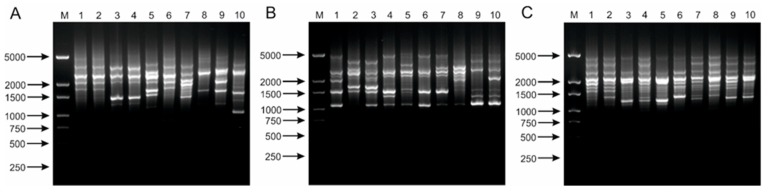
(GTG)_5_-PCR patterns of 30 strains belonging to *L. casei* group. Analysis of the discriminatory power of the procedure applied was performed for 10 strains of *L. casei* (**A**)—M, DNA molecular marker; 1, LMG 6904; 2, LMG 23516; 3, LMG 24099; 4, LMG 24102; 5, JCM 2120; 6, JCM 8129; 7, JCM 8608; 8, JCM 8677; 9, JCM 20024; 10, LMG 17315; 10 strains of *L. paracasei* (**B**)—M, DNA molecular marker; 1, LMG 13087; 2, LMG 9193; 3, LMG 9438; 4, LMG 11459; 5, LMG 11961; 6, LMG 12164; 7, LMG 19719; 8, JCM 1163; 9, LMG 9191; 10, JCM 20315; and 10 strains of *L. rhamnosus* (**C**)—M, DNA molecular marker; 1, LMG 6400; 2, LMG 8153; 3, LMG 10768; 4, LMG 10772; 5, LMG 12166; 6, LMG 18030; 7*,* LMG 23304; 8, LMG 23536; 9, LMG 23550; 10, LMG 25881.

An alternative solution to RAPD-PCR, characterized by both high differential strength and satisfactory reproducibility, is the amplification of repetitive regions dispersed in bacterial genomes [48,49]. In our research, we used ERIC, (GTG)_5_ and BOXA1R primers (Table S1) for this purpose. The results obtained showed that for the strains belonging to *L. casei,* the highest differential strength was characterized by the (GTG)_5_-PCR reaction. In this reaction, four different profiles of PCR products were observed for the tested strains (Figure 4A). In the case of other primers (BOX and ERIC), identical profiles were obtained for LMG 6904, LMG 23516 and JCM 8129, as well as for LMG 24099 and LMG 24102 (Figures S6A and S7A). Additionally, in the BOX-PCR reaction, it was difficult to differentiate strains LMG 24099, LMG 24102 and LMG 17315 (Figure S6A).

As for *L. casei*, by far the best results for *L. paracasei* strains were observed when analyzing the electrophoretic separation of PCR products obtained with the (GTG)_5_ primer. With this procedure, it was possible to differentiate 10 *L. paracasei* strains used in this study (also belonging to *L. paracasei subsp. tolerans* species) (Figure 4B). It was problematic obtaining strain-specific results for LMG 9193 and JCM 2120, which were originally classified as *L. casei*. The other two reaction systems (ERIC-PCR and BOX-PCR) showed much lower differentiation potential for the tested *L. paracasei* strains (Figures S6B and S7B).

The final species tested in this part of the study was *L. rhamnosus*. Both ERIC-PCR and GTG_5_-PCR (Figure 4C and Figure S7C) proved to be effective differentiation methods for the tested strains. The BOX-PCR procedure (Figure S6C) proved to be less effective in strain differentiation. The results obtained with ERIC primers showed that the only strains for which strain-specific profiles of PCR products could not be obtained were LMG 10768 and LMG 12166. Very similar profiles were also obtained for LMG 10772 and JCM 8608 (*L. casei*). In the case of the GTG_5_-PCR method, very similar PCR product distributions were obtained for the tested strains, with lengths of approximately 1000-5000 bp. Despite the high similarity of the observed results, detailed analysis made it possible to distinguish all tested isolates. The most similar DNA profiles for this species were obtained for strains LMG 10772 and LMG 23304.

#### 2.3.3. Amplified Length Polymorphism Analysis

The final method used to differentiate the *L. casei* group at the strain level was the DNA fingerprinting technique called amplified length polymorphism analysis (AFLP). This method is based on the analysis of the length of PCR products derived from amplified restriction fragments of a total digest of genomic DNA. As with previous methods, this procedure does not require knowledge of the genomic sequence of the microorganisms tested [50]. Numerous studies have shown that this method is very useful in genotyping bacteria, including those belonging to the *L. casei* group [24,51,52,53]. Compared to the previously described methods, this technique is characterized by both higher reproducibility and high differential strength, even at the strain level [54]. The results obtained in this study also showed a higher differential strength in the AFLP methods compared to RAPD-PCR and Rep-PCR techniques. All obtained DNA profiles, which were analyzed within the range of 100 to 450 bp, were characterized by a unique, strain-specific pattern of amplification products. The obtained DNA profiles for particular species are shown in Figures S8–S10. Detailed analysis showed that, even for strains which in previous tests generated identical or very similar DNA profiles, strain-specific electrophoretic patterns were obtained with the AFLP method. Good examples of this are the strains belonging to *L. casei*; LMG 6904, LMG 23516 and JCM 8129, as well as LMG 24099 and LMG 24102, for which identical results were obtained using RAPD-PCR primers and Rep-PCR (GTG_5_, ERIC and BOXA1R). In the case of the AFLP technique, even for these strains, amplification products were obtained which made it possible to easily differentiate them from other isolates belonging to *L. casei* (Figure S8). Similar results were also obtained for *L. paracasei* strains (including *L. paracasei subsp. tolerans*) (Figure S9), as well as microorganisms belonging to *L. rhamnosus* (Figure S10). Summarizing this stage of the research, it should be stated that, among the techniques classified as DNA fingerprinting methods, the AFLP method showed by far the highest potential for differentiation of bacteria classified under the *L. casei* group.

Using applied AFLP, it was possible to obtain characteristic DNA profiles for each of the tested strains. On the other hand, compared to RAPD-PCR and Rep-PCR techniques, this method is much more time-consuming and requires more advanced hardware and software for analysis. Furthermore, the large number of steps, including restriction digestion, adapter ligation, double amplification and electrophoretic analysis, also foster technical errors, which may affect the reproducibility of results, especially when genotyping large numbers of new isolates. Therefore, in some experiments where differential strength is not essential, the use of previously tested methods such as RAPD-PCR or Rep-PCR also seems appropriate. The simplicity of these analyses and their relatively low unit cost is an important advantage, especially in the initial phase of studies where large numbers of new strains are isolated from a given source. In this case, on the basis of the obtained results, it can be concluded that for the microorganisms belonging to the *L. casei* group, after the AFLP technique, the RAPD method using M13 primer and the GTG_5_-PCR technique showed the highest efficiency.

### 2.4. Proteomic Procedures—SDS-PAGE Profiles and MALDI-TOF MS

Numerous scientific reports present the possibility of using proteomic methods for the differentiation and identification of microorganisms. The described procedures include both relatively simple to perform electrophoretic analyses, and methods based on mass spectrometry [55,56].

In the final part of this study, electrophoretic analysis of proteins under denaturing conditions, and matrix-assisted laser desorption ionization time-of-flight mass spectrometry were used to differentiate and identify *L. casei* bacteria. During SDS-PAGE analysis of proteins, one important issue with regard to bacterial differentiation at the strain level is the proper preparation of protein samples. In the case of microorganisms belonging to the *L. casei* group, which have different optimum bacterial growth temperatures, the microorganisms being examined should initially be divided at the culture stage. Most commercial electrophoresis kits only allow the analysis of small amounts of samples in a single gel, which may also present a source of difficulty in analyzing the results obtained. Previous studies have shown that the protein profiles obtained are typically very complex, which may limit the usefulness of such results for taxonomic identification [13]. On the other hand, this method seems to be a good solution when the homogeneity of a bacterial culture needs to be confirmed, e.g., when a given strain generates more than one type of colony morphology [57].

**Figure 5:**
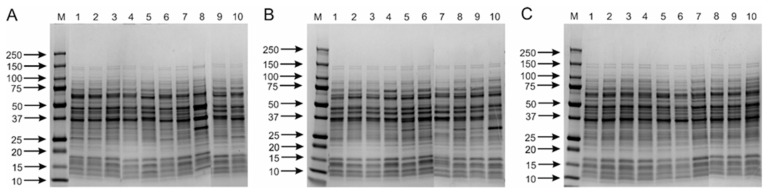
SDS-PAGE profiles of whole cell proteins obtained for all tested lactobacilli belong to *L. casei* group. Analysis of the discriminatory power of this technique was performed for 10 strains of *L. casei* (**A**)—M, protein molecular weight marker; 1, LMG 6904; 2, LMG 23516; 3, LMG 24099; 4, LMG 24102; 5, JCM 2120; 6, JCM 8129; 7, JCM 8608; 8, JCM 8677; 9, JCM 20024; 10, LMG 17315; 10 strains of *L. paracasei* (**B**)—M, protein molecular weight marker; 1, LMG 13087; 2, LMG 9193; 3, LMG 9438; 4, LMG 11459; 5, LMG 11961; 6, LMG 12164; 7, LMG 19719; 8, JCM 1163; 9, LMG 9191; 10, JCM 20315; and 10 strains of *L. rhamnosus* (**C**)—M, protein molecular weight marker; 1, LMG 6400; 2, LMG 8153; 3, LMG 10768; 4, LMG 10772; 5, LMG 12166; 6, LMG 18030; 7*,* LMG 23304; 8, LMG 23536; 9, LMG 23550; 10, LMG 25881.

For the microorganisms tested in this study, protein distributions in the range ~10 to 150 kDa were analyzed. For six strains belonging to the species *L. casei*, very similar or identical protein distributions were obtained (Figure 5A). The procedure used made it possible to identify the tested microorganisms at the species level. Analyzing both the distribution of protein bands, as well as their intensity, it was difficult to observe significant differences between individual strains of this species. Similar results were obtained for *L.rhamnosus* (Figure 5C). In this case, it was possible to identify the species unambiguously, and slight differences in obtained protein profiles were observed in some isolates, which made it possible to differentiate at the strain level. For *L. paracasei*, on the basis of the most intense protein bands, it was possible to confirm the species affiliation of the tested strains (Figure 5B). Compared to *L. casei* and *L. rhamnosus*, the obtained protein distributions were more varied. Particular differences in the distribution of bands and their intensity were observed in the range of ~20 to 40 kDa. Interestingly, despite lower culture temperature for two strains belonging to the *L. paracasei* subsp. *tolerans* subspecies, if not identical, then very similar results were obtained in comparison to other *L. paracasei* strains.

When collectively analyzing the results obtained by another method—MALDI-TOF MS, 10 out of 30 tested strains gave BioTyper log(score) values above 2.3. For the subsequent 17 strains, the value of this parameter was in the range of 2.0 to 2.3. For two tested isolates, a result between 1.7 and 2 was obtained, and for one strain, a result below 1.7 (Table 2). Analyzing the obtained results for particular species of the *L. casei* group, it is immediately evident that the highest BioTyper log(score) values were observed for *L. paracasei* strains (2.186 to 2.473). Noticeably lower results were obtained for *L. rhamnosus* (2.052 to 2.282). A surprising exception was *L. rhamnosus* strain LMG 12166, for which, despite several repetitions, a maximum result of 1.541 was obtained. Interestingly, with the use of previous methods, it was possible to clearly classify this strain as a *L. rhamnosus* strain. In the case of strains which were originally classified as *L. casei*, in six cases the affiliation to this species was confirmed (scores from 1.922 to 2.199). As in the PCR based assays, strains JCM 2120 and JCM 20024 were classified as *L. paracasei,* and JCM 8608 as *L. rhamnosus*. For JCM 8677, a result of 1.83 was obtained, which suggested taxonomic affiliation to *L. crispatus*. This was in accordance with previous results, from which it could be concluded that this strain is not a member of the *L. casei* group.

In conclusion, the results obtained confirmed the effectiveness of both proteomic methods used in the identification of *L. casei* strain species. The MALDI-TOF MS method seems to be particularly noteworthy, and may prove an interesting alternative to procedures using PCR. Despite the high cost of equipment, the identification procedure itself is quick and easy to perform, and the results obtained are easy to interpret. In addition to taxonomic classification of microorganisms, this method also offers the possibility to identify potential bacterial contamination, which is an undeniable advantage [14,27,58,59].

## 3. Materials and Methods

### 3.1. Bacterial Strains and Culture Conditions

For this study we used 30 strains of *L. casei* group bacteria from two international collections of microorganisms—the Japan Collection of Microorganisms (JCM) and Belgian Coordinated Collections of Microorganisms (BCCM) (Table 1). Bacteria were cultured in MRS medium (Oxoid) at 30 °C or 37 °C according to the instructions provided by the source collection of microorganisms.

### 3.2. DNA Isolation

DNA samples were prepared from a 1 mL aliquot of 24 h culture using the commercial Genomic Mini AX BACTERIA+ kit (A&A Biotechnology, Gdynia, Poland). The isolation was carried out according to the manufacturer’s protocol. Next, DNA was dissolved in Tris buffer (10 mM Tris HCl, pH 8.5) and the concentration of nucleic acids was estimated using a Nanodrop 2000c spectrophotometer (Thermo Fisher Scientific, Waltham, USA). The purified DNA samples were then diluted to 25 ng/µL and the final concentration was verified using a Qubit 4 fluorometer (Thermo Fisher Scientific). Samples were stored at −20 °C until later use.

### 3.3. Polymerase Chain Reaction Conditions

PCR reactions were conducted in a T100 thermocycler (Biorad, Hercules, USA), using reagents from Thermo Fisher Scientific and EURx. Primers, reagent concentrations and thermal conditions for standard reactions, RFLP-PCR, Multiplex-PCR, RAPD-PCR and rep-PCR are presented in Table S1. In the case of RFLP-PCR, the PCR products were digested with selected restriction enzymes (ApoI, HaeIII and MseI) under a protocol provided by the manufacturer (Thermo Fisher Scientific) (Table S1).

### 3.4. Electrophoretic Separation and Data Analysis

Electrophoretic separations of DNA were conducted in gels prepared from agarose (EURx, Gdansk, Poland), Tris-acetate-EDTA buffer (TAE) and ethidium bromide (0.5 µg/mL), using Electrophoresis Sub-Cell and Wide Mini-Sub Cell GT systems (Biorad). The PCR products were visualized under UV light (Gel Doc XR+ gel documentation system, Bio-Rad) and the length of DNA molecules was evaluated against a DNA molecular marker—GeneRuler 100 bp DNA Ladder (Thermo Fisher Scientific), O’GeneRuler 100 bp Plus DNA Ladder (Thermo Fisher Scientific) and Perfect Plus Molecular Weight Quantitative DNA Ladder (EURx). The results were analyzed using Image Lab Software (Biorad), Quantity One 1-D Analysis System (Biorad) and PyElph 1.4 [60].

### 3.5. Real-time PCR and High-Resolution Melting Analysis

The HRM-PCR reaction was carried out with two set of primers, based on the *spxB* and *groEL* genes (Table S1) [39,40]. PCR reactions were performed in 15-μL solutions containing 7.5 µL of 2× SsoFast EvaGreen Supermix (Biorad), 0.5 µM or 0.25 µM of *poxcDNAFw/poxPromRv and* GroHRM-F/GroHRM-R primers, and 15 ng of bacterial DNA. Amplification was conducted using the CFX Connect thermocycler (Biorad) as follows: 1 min at 98 °C for initial denaturation and 50 cycles of 15 sec at 95 °C and an annealing/extension step at 60 °C for 1 min (*poxcDNAFw and poxPromRv primers);* 1 min at 98 °C for initial denaturation and 45 cycles of 30 s at 95 °C, an annealing step at 59 °C for 20 sec and 1 sec at 72 °C for extension (GroHRM-F/GroHRM-R primers). After amplification, melting curves of the PCR products were determined by monitoring fluorescence from 65 to 95 °C, with temperature increments of 0.2 °C. The results were analysed with Biorad CFX Manager and uAnalyzeSM v. 2.0 [39,61].

### 3.6. Amplified Fragment Length Polymorphism Analysis

DNA was first digested with EcoRI and MseI (Thermo Fisher Scientific) enzymes. The 20 µL reaction mixture consisted of 0.5 µL of the enzyme EcoRI (5 U/µL), 4 µl of Tango buffer and 200 ng of bacterial DNA and nuclease-free water. The reaction mixture was incubated at 37 °C for 2 h and 0.5 µL of MseI (5 U/µL), 2 µL of Tango buffer and 7.5 µL of H_2_O were added to each sample. The samples were incubated at 65 °C for a further 2 h.

Next, ligation mixes were prepared which contained 1µl T4 DNA ligase (1U/µL) (Thermo Fisher Scientific), 2 µL 1× T4 DNA ligase buffer (Thermo Fisher Scientific), 1 µL 5 µM of EcoRI adaptors (5’-CTCGTAGACTGCGTACC-3’ and 5’-AATTGGTACGCAGTCTAC-3’), 1 µL 50 µM of MseI adaptors (5’-GACGATGAGTCCTGAG-3’ and 5’-TACTCAGGACTCAT-3’), 30 µL of reaction mixture after restriction digestion and 5 µL of nuclease-free water. Before being added to the ligation mixture, the adaptors were incubated at 95 °C for 3 min and then at room temperature for 10 min. Ligation was carried out at 4 °C for approximately 18 h.

Next, pre-selective PCR was conducted using EcoRI+0—5’-GACTGCGTACCAATTC-3’ and MseI+0—5’-GATGAGTCCTGAGTAA-3’ primers. The 40 µL reaction mixture consisted of 20 µL DreamTaq PCR Master Mix (2×) (Thermo Fisher Scientific), 10 µL of 1:9 diluted ligation mixture, and EcoRI+0 and MseI+0 primers, having a final concentration of 1 µM. The program consisted of pre-denaturation (94 °C, 5 min), and 23 cycles, which included denaturation (94 °C, 30 s), annealing (56 °C, 30 s) elongation (72 °C, 30 s), and final elongation (60 °C, 30 min). The resulting reaction mixture was then diluted again (1:9) and used for selective PCR using the fluorescently-labelled EcoRI-CAT primer—5’-FAM-GACTGCGTACCAATTCCAT-3’ and the specific primer MseI-CTT—5’-GATGAGTCCTGAGTAACTT-3’. The final reaction volume was 20 µL and contained 10 µL of DreamTaq PCR Master Mix (2×) (Thermo Scientific), 2.5 µL diluted generic PCR reaction mixture, 0.25 µM of EcoRI-CAT primer and 1 µM of MseI-CTT primer. Amplification consisted of pre-denaturation carried out at 94 °C for 5 min, and 30 repetitions comprising denaturation (94 °C, 30 s), annealing (56 °C, 30 s) and elongation (72 °C, 30 s). The products were analyzed in a capillary sequencer, the 3730xl DNA Analyzer (Life Technologies, Carlsbad, USA) by Genomed. The results were analysed using Peak Scanner software (Thermo Fisher Scientific).

### 3.7. SDS-PAGE Fingerprinting

For cell-free extract preparation, lactobacilli were grown anaerobically in 10 mL of MRS medium. Bacterial cultures were incubated at 37 °C for 24 h, with the exception of two *Lactobacillus paracasei* subsp. *tolerans* strains, which were grown at 30 °C. Then, cells were harvested by centrifugation at 7,142× *g* for 10 min at 4 °C. The cell pellets were washed twice in PBS buffer and resuspended in 1000 µL of 0.1 M sodium-phosphate buffer (pH 7.0). Cells were then disrupted by sonication for 5 min with constant cooling, followed by centrifugation at 16,100× *g* for 10 min at 4 °C. The supernatant was stored at –20 °C. Protein concentrations were measured using a Qubit 4 fluorometer (Thermo Fisher Scientific), and the samples (~10 µg) were tested with SDS-PAGE. The protein samples were separated in 4–20% precast polyacrylamide gel (Biorad) in a Mini-Protean Tetra Cell (Biorad). Proteins were visualized by staining with Coomassie Brilliant Blue R-250. The results were analyzed using a Gel Doc XR+ gel documentation system (Biorad).

### 3.8. MALDI-TOF Mass Spectrometry

Two to five single bacterial colonies were used for the preparation of each sample. Bacterial suspensions were prepared in 300 µL of distilled water, then fixed by the addition of 900 µL absolute ethanol. Then, the samples were centrifuged at 13,000× *g* for 5 min, and the supernatants were removed. Lysates were made by adding 50 µL 70% formic acid and 50 µL acetonitrile to each bacterial pellet. Following centrifugation (13,000× *g*, 2 min), the supernatants were transferred to fresh tubes, and 1 µL of each supernatant containing bacterial lysate was transferred onto a spot of the 384 MTP AnchorChip™ T F stainless steel MALDI target plate (Bruker Daltonics, Billerica, USA). The samples were overlaid with 1 µL of MALDI matrix and air-dried.

The measurements were conducted using a Bruker Daltonics UltrafleXtreme spectrometer. Spectra were recorded in the positive linear mode for a mass range of 2000 to 20000 Da (laser frequency 200 Hz; ion source voltage one, 25 kV; ion source voltage two, 23.5 kV; lens voltage, 6.0 kV). Each spectrum was obtained by averaging 1500 laser shots acquired from three spot positions, under the control of flexControl software 3.4 (Bruker Daltonics). Prior to the analysis, the spectra were calibrated using a bacterial test standard (Bruker Daltonics). For the bacterial identification, Biotyper 3.1 software (Bruker Daltonics) and a database containing 6904 entries were applied. The following score values were used: less than 1.7—identification not reliable, 1.7–2.0—probable genus identification, 2.0–2.3 secure genus identification and probable species identification, and more than 2.3—highly probable species identification.

## 4. Conclusions

In this study, for specific identification of strains belonging to the *L. casei* group, several of the most commonly used procedures have been used, in which it is not necessary to know the genome sequences of the isolates tested. Despite the rapid development of techniques based on direct analysis of multiple gene sequences (MLST), or even entire genomes, in some cases, the use of these procedures is still justified. In particular, when a large group of new isolates is being tested, and where factors such as the cost of a single analysis, realization time and availability of advanced equipment are important issues, classical identification and differentiation techniques still seem to be a highly practical and effective solution. On the other hand, it should be emphasised that further development of both the next-generation sequencing and data analysis methods will allow for the design of effective approaches for the unambiguous identification of individual bacterial strains. Currently, such analyses as average nucleotide identity (ANI), digital DNA-DNA hybridization or tetranucleotide usage patterns (TETRA) are becoming increasingly popular in bacterial genotyping. The application of these methods in taxonomy of *L.casei* group bacteria has been described in detail in Wuyts et. al. [5] and Huang et al. [14].

Using the procedures described in this study, the original phylogenetic affiliation was confirmed for 26 of the 30 strains analyzed. In the case of three strains (JCM 2120, JCM 20024 and JCM 8608), which belonged to the *L. casei* group, these isolates were wrongly classified as *L. casei* species. Interestingly, strain JCM 8677 did not belong to any of the three target species. Summarizing the results obtained, it can be concluded that, of the methods used to identify *L. casei* group bacteria at the species level, the most effective were multiplex PCR and MALDI-TOF MS. These procedures were characterized by quick performance, a relatively low level of difficulty and uncomplicated analysis of results. The remaining methods, in many cases, also showed efficacy in genotyping, therefore they may be a valuable alternative in cases in which multiplex PCR and MALDI-TOF MS do not produce conclusive results.

When analyzing methods for microbial differentiation at the strain level, AFLP proved to be by far the most effective procedure. Nevertheless, due to the rather complex procedure, especially for preliminary studies involving large numbers of new isolates, techniques based on rep-PCR may also be a viable solution. Despite the lower differential strength compared to AFLP, rep-PCR methods are much easier to perform technically and do not require sophisticated equipment.

In conclusion, taking into account both the specifics of the conducted tests as well as the availability of specific laboratory equipment, we hope that the results presented by us will facilitate the easy selection of methods for identifying new strains belonging to the *L. casei* group. Moreover, due to the isolation of new species similar to the bacteria described in this paper, we believe that the presented research will contribute to the development of new, even more effective procedures, which will enable identification not only at the species level but also at the subspecies and strain levels.

## Figures and Tables

**Table 1:** Results obtained in various PCR reactions using 30 bacterial strains of *L. casei* group. Detailed information on the PCR reaction conditions and primer sequences is provided in Table S1.

Bacterial Strain	PCR Reaction (Primer Names)													
	Genus-Specific LbLMA1-rev/R16-1	Genus-Specific TUF-1/TUF-2	*L. casei* Group-Specific LCgprpoA-F2/LCgprpoA-R2	*L. casei*-Specific Y2/casei (W1)	*L. paracasei-*Specific Y2/para (W2)	*L. rhamnosus*-Specific Y2/rham (W3)	*L. zeae*-Specific Y2/zeae (D1)	*L. zeae-*Specific SpeOPT16zeae-F SpeOPT16zeae-R	*L. rhamnosus*-Specific SpeOPT14rhaF SpeOPT14rha-R	Subspecies-Specific SpeOPT11tolF SpeOPT11tol-R	Multiplex CPR/CAS, PAR, RHA (Number of Amplicons)	Multiplex CZfor, PC2a, RHfor/CPRrev PRODUCT Length (bp)	HRM—*spxB* *poxcDNAFw/poxPromRv* Melting Temp. (°C)	HRM—*groEL* GroHRM-F/GroHRM-R Melting Temp. (°C)
*Lactobacillus casei*														
*L. casei* LMG 6904	+	+	+	+	- (ns *)	- (ns)	+	-	-	-	+ (4)	+ (666)	81.70	78.40
*L. casei* LMG 23516	+	+	+	+	- (ns)	- (ns)	+	-	-	-	+ (4)	+ (666)	81.60	78.40
*L. casei* LMG 24099	+	+	+	+	- (ns)	- (ns)	+	-	-	-	+ (4)	+ (666)	81.60	78.00
*L. casei* LMG 24102	+	+	+	+	-	- (ns)	+	-	-	-	+ (4)	+ (666)	81.60	78.10
*L. casei* JCM 2120	+	+	+	- (ns)	+	-	-	-	-	-	+ (2)	+ (253)	81.20	79.50
*L. casei* JCM 8129	+	+	+	+	-	- (ns)	+	-	-	-	+ (4)	+ (666)	81.60	78.40
*L. casei* JCM 8608	+	+	+	- (ns)	-	+	-	-	+	-	+ (1)	-	82.80	79.80
*L. casei* JCM 8677	+	+	-	- (ns)	-	-	-	-	-	-	-	-	-	-
*L. casei* JCM 20024	+	+	+	- (ns)	+	-	- (ns)	-	-	-	+ (2)	+ (253)	81.20	79.10
*L. casei* LMG 17315	+	+	+	+	-	- (ns)	+	+	-	-	+ (4)	+ (666)	81.60	78.30
*Lactobacillus paracasei*														
*L. paracasei* LMG 13087	+	+	+	- (ns)	+	-	- (ns)	-	-	-	+ (2)	+ (253)	81.60	79.50
*L. paracasei* LMG 9193	+	+	+	- (ns)	+	- (ns)	- (ns)	-	-	-	+ (2)	+ (253)	81.30	79.50
*L. paracasei* LMG 9438	+	+	+	- (ns)	+	- (ns)	- (ns)	-	-	-	+ (2)	+ (253)	81.20	79.50
*L. paracasei* LMG 11459	+	+	+	- (ns)	+	- (ns)	- (ns)	-	-	-	+ (2)	+ (253)	81.30	79.40
*L. paracasei* LMG 11961	+	+	+	- (ns)	+	- (ns)	- (ns)	-	-	-	+ (2)	+ (253)	81.50	79.20
*L. paracasei* LMG 12164	+	+	+	- (ns)	+	- (ns)	- (ns)	-	-	-	+ (2)	+ (253)	81.30	79.50
*L. paracasei* LMG 19719	+	+	+	- (ns)	+	- (ns)	- (ns)	-	-	-	+ (2)	+ (253)	81.50	79.50
*L. paracasei* JCM 1163	+	+	+	- (ns)	+	- (ns)	-	-	-	-	+ (2)	+ (253)	81.50	79.20
*L. paracasei* LMG 9191	+	+	+	- (ns)	+	- (ns)	- (ns)	-	-	-	+ (2)	+ (253)	81.20	79.20
*L. paracasei* JCM 20315	+	+	+	- (ns)	+	- (ns)	- (ns)	-	-	+	+ (2)	+ (253)	81.20	79.20
*Lactobacillus rhamnosus*														
*L. rhamnosus* LMG 6400	+	+	+	-	-	+	-	-	+	-	+ (1)	+ (800)	83.40	79.80
*L. rhamnosus* LMG 8153	+	+	+	-	-	+	-	-	+	-	+ (1)	+ (800)	83.20	79.80
*L. rhamnosus* LMG 10768	+	+	+	-	-	+	-	-	+	-	+ (1)	+ (800)	82.70	79.80
*L. rhamnosus* LMG 10772	+	+	+	-	- (ns)	+	-	-	+	-	+ (1)	+ (800)	82.70	79.70
*L. rhamnosus* LMG 12166	+	+	+	-	-	+	-	-	+	-	+ (1)	+ (800)	82.80	79.80
*L. rhamnosus* LMG 18030	+	+	+	-	-	+	-	-	+	-	+ (1)	+ (800)	83.30	79.00
*L. rhamnosus* LMG 23304	+	+	+	-	- (ns)	+	-	-	+	-	+ (1)	+ (800)	82.80	79.80
*L. rhamnosus* LMG 23536	+	+	+	-	-	+	-	-	+	-	+ (1)	+ (800)	83.30	79.50
*L. rhamnosus* LMG 23550	+	+	+	-	-	+	-	-	+	-	+ (1)	+ (800)	83.20	79.80
*L. rhamnosus* LMG 25881	+	+	+	-	-	+	-	-	+	-	+ (1)	+ (800)	82.80	79.70

* non-specific PCR product.

**Table 2:** Species confirmation of bacterial strains belonging to the *L. casei* group using MALDI-TOF MS. All experiments were performed in duplicate, the highest log(score) is shown in the table.

No.	Species	Strain	Highest Biotyper log(score)	MALDI-TOF MS
1	*L. casei*	LMG 6904	1.922	*L. casei*
2	*L. casei*	LMG 23516	2.06	*L. casei*
3	*L. casei*	LMG 24099	2.024	*L. casei*
4	*L. casei*	LMG 24102	2.199	*L. casei*
5	*L. casei*	JCM 2120	2.462	*L. paracasei*
6	*L. casei*	JCM 8129	2.014	*L. casei*
7	*L. casei*	JCM 8608	2.142	*L. rhamnosus*
8	*L. casei*	JCM 8677	1.83	*L. crispatus*
9	*L. casei*	JCM 20024	2.338	*L. paracasei*
10	*L. casei*	LMG 17315	2.18	*L. casei*
11	*L. paracasei*	LMG 13087	2.252	*L. paracasei*
12	*L. paracasei*	LMG 9193	2.308	*L. paracasei*
13	*L. paracasei*	LMG 9438	2.186	*L. paracasei*
14	*L. paracasei*	LMG 11459	2.453	*L. paracasei*
15	*L. paracasei*	LMG 11961	2.404	*L. paracasei*
16	*L. paracasei*	LMG 12164	2.381	*L. paracasei*
17	*L. paracasei*	LMG 19719	2.394	*L. paracasei*
18	*L. paracasei*	JCM 1163	2.384	*L. paracasei*
19	*L. paracasei*	LMG 9191	2.473	*L. paracasei*
20	*L. paracasei*	JCM 20315	2.369	*L. paracasei*
21	*L. rhamnosus*	LMG 6400	2.205	*L. rhamnosus*
22	*L. rhamnosus*	LMG 8153	2.223	*L. rhamnosus*
23	*L. rhamnosus*	LMG 10768	2.183	*L. rhamnosus*
24	*L. rhamnosus*	LMG 10772	2.052	*L. rhamnosus*
25	*L. rhamnosus*	LMG 12166	1.541	*-*
26	*L. rhamnosus*	LMG 18030	2.065	*L. rhamnosus*
27	*L. rhamnosus*	LMG 23304	2.282	*L. rhamnosus*
28	*L. rhamnosus*	LMG 23536	2.061	*L. rhamnosus*
29	*L. rhamnosus*	LMG 23550	2.166	*L. rhamnosus*
30	*L. rhamnosus*	LMG 25881	2.24	*L. rhamnosus*

## Supplementary Materials

Supplementary materials can be found at https://www.mdpi.com/1422-0067/21/8/2694/s1.
