# Supplementary material for: Molecular Routes to Specific Identification of the Lactobacillus Casei Group at the Species, Subspecies and Strain Level
Source: Int J Mol Sci. 2020 Apr 13;21(8):2694. doi: 10.3390/ijms21082694 (PMC7216162; doi:10.3390/ijms21082694)
Supplement: Supplementary file 1 [file ijms-21-02694-s001.zip › ijms-752512-Proofdone sup/supplementary materials.docx]

Legend

**Figure S1**

RFLP patterns generated from restriction analysis of 16S rRNA amplicon (~1500 bp) of 30 strains belong to *L. casei* group using MseI restrictase.

Analysis of the discriminatory power of the procedure applied was performed for 10 strains of *L. casei* (A) - M, DNA molecular marker; 1, LMG 6904; 2, LMG 23516; 3, LMG 24099; 4, LMG 24102; 5, JCM 2120; 6, JCM 8129; 7, JCM 8608; 8, JCM 8677; 9, JCM 20024; 10, LMG 17315; 10 strains of *L. paracasei* (B) - M, DNA molecular marker; 1, LMG 13087; 2, LMG 9193; 3, LMG 9438; 4, LMG 11459; 5, LMG 11961; 6, LMG 12164; 7, LMG 19719; 8, JCM 1163; 9, LMG 9191; 10, JCM 20315; and 10 strains of *L. rhamnosus* (C) - M, DNA molecular marker; 1, LMG 6400; 2, LMG 8153; 3, LMG 10768; 4, LMG 10772; 5, LMG 12166; 6, LMG 18030; 7*,* LMG 23304; 8, LMG 23536; 9, LMG 23550; 10, LMG 25881.

**Figure S2**

RFLP patterns generated from restriction analysis of *dnaK* amplicon (~1000 bp) of 30 strains belong to *L. casei* group using ApoI restrictase.

Analysis of the discriminatory power of the procedure applied was performed for 10 strains of *L. casei* (A) - M, DNA molecular marker; 1, LMG 6904; 2, LMG 23516; 3, LMG 24099; 4, LMG 24102; 5, JCM 2120; 6, JCM 8129; 7, JCM 8608; 8, JCM 8677; 9, JCM 20024; 10, LMG 17315; 10 strains of *L. paracasei* (B) - M, DNA molecular marker; 1, LMG 13087; 2, LMG 9193; 3, LMG 9438; 4, LMG 11459; 5, LMG 11961; 6, LMG 12164; 7, LMG 19719; 8, JCM 1163; 9, LMG 9191; 10, JCM 20315; and 10 strains of *L. rhamnosus* (C) - M, DNA molecular marker; 1, LMG 6400; 2, LMG 8153; 3, LMG 10768; 4, LMG 10772; 5, LMG 12166; 6, LMG 18030; 7*,* LMG 23304; 8, LMG 23536; 9, LMG 23550; 10, LMG 25881.

**Figure S3**

Randomly amplified polymorphic DNA (RAPD)-PCR patterns obtained with 80A_RAPD primer for 30 lactobacilli belong to *L. casei* group.

Analysis of the discriminatory power of the procedure applied was performed for 10 strains of *L. casei* (A) - M, DNA molecular marker; 1, LMG 6904; 2, LMG 23516; 3, LMG 24099; 4, LMG 24102; 5, JCM 2120; 6, JCM 8129; 7, JCM 8608; 8, JCM 8677; 9, JCM 20024; 10, LMG 17315; 10 strains of *L. paracasei* (B) - M, DNA molecular marker; 1, LMG 13087; 2, LMG 9193; 3, LMG 9438; 4, LMG 11459; 5, LMG 11961; 6, LMG 12164; 7, LMG 19719; 8, JCM 1163; 9, LMG 9191; 10, JCM 20315; and 10 strains of *L. rhamnosus* (C) - M, DNA molecular marker; 1, LMG 6400; 2, LMG 8153; 3, LMG 10768; 4, LMG 10772; 5, LMG 12166; 6, LMG 18030; 7*,* LMG 23304; 8, LMG 23536; 9, LMG 23550; 10, LMG 25881.

**Figure S4**

Randomly amplified polymorphic DNA (RAPD)-PCR patterns obtained with 80C_RAPD_OPT-14 primer for 30 lactobacilli belong to *L. casei* group.

Analysis of the discriminatory power of the procedure applied was performed for 10 strains of *L. casei* (A) - M, DNA molecular marker; 1, LMG 6904; 2, LMG 23516; 3, LMG 24099; 4, LMG 24102; 5, JCM 2120; 6, JCM 8129; 7, JCM 8608; 8, JCM 8677; 9, JCM 20024; 10, LMG 17315; 10 strains of *L. paracasei* (B) - M, DNA molecular marker; 1, LMG 13087; 2, LMG 9193; 3, LMG 9438; 4, LMG 11459; 5, LMG 11961; 6, LMG 12164; 7, LMG 19719; 8, JCM 1163; 9, LMG 9191; 10, JCM 20315; and 10 strains of *L. rhamnosus* (C) - M, DNA molecular marker; 1, LMG 6400; 2, LMG 8153; 3, LMG 10768; 4, LMG 10772; 5, LMG 12166; 6, LMG 18030; 7*,* LMG 23304; 8, LMG 23536; 9, LMG 23550; 10, LMG 25881.

**Figure S5**

Randomly amplified polymorphic DNA (RAPD)-PCR patterns obtained with 80D_RAPD_OPA-18 primer for 30 lactobacilli belong to *L. casei* group.

Analysis of the discriminatory power of the procedure applied was performed for 10 strains of *L. casei* (A) - M, DNA molecular marker; 1, LMG 6904; 2, LMG 23516; 3, LMG 24099; 4, LMG 24102; 5, JCM 2120; 6, JCM 8129; 7, JCM 8608; 8, JCM 8677; 9, JCM 20024; 10, LMG 17315; 10 strains of *L. paracasei* (B) - M, DNA molecular marker; 1, LMG 13087; 2, LMG 9193; 3, LMG 9438; 4, LMG 11459; 5, LMG 11961; 6, LMG 12164; 7, LMG 19719; 8, JCM 1163; 9, LMG 9191; 10, JCM 20315; and 10 strains of *L. rhamnosus* (C) - M, DNA molecular marker; 1, LMG 6400; 2, LMG 8153; 3, LMG 10768; 4, LMG 10772; 5, LMG 12166; 6, LMG 18030; 7*,* LMG 23304; 8, LMG 23536; 9, LMG 23550; 10, LMG 25881.

**Figure S6**

BOX-PCR DNA profiles obtained for 30 strains belonging to *L. casei* group.

Analysis of the discriminatory power of this procedure was performed for 10 strains of *L. casei* (A) - M, DNA molecular marker; 1, LMG 6904; 2, LMG 23516; 3, LMG 24099; 4, LMG 24102; 5, JCM 2120; 6, JCM 8129; 7, JCM 8608; 8, JCM 8677; 9, JCM 20024; 10, LMG 17315; 10 strains of *L. paracasei* (B) - M, DNA molecular marker; 1, LMG 13087; 2, LMG 9193; 3, LMG 9438; 4, LMG 11459; 5, LMG 11961; 6, LMG 12164; 7, LMG 19719; 8, JCM 1163; 9, LMG 9191; 10, JCM 20315; and 10 strains of *L. rhamnosus* (C) - M, DNA molecular marker; 1, LMG 6400; 2, LMG 8153; 3, LMG 10768; 4, LMG 10772; 5, LMG 12166; 6, LMG 18030; 7*,* LMG 23304; 8, LMG 23536; 9, LMG 23550; 10, LMG 25881.

**Figure S7**

ERIC-PCR DNA profiles obtained for 30 strains belonging to *L. casei* group.

Analysis of the discriminatory power of this procedure was performed for 10 strains of *L. casei* (A) - M, DNA molecular marker; 1, LMG 6904; 2, LMG 23516; 3, LMG 24099; 4, LMG 24102; 5, JCM 2120; 6, JCM 8129; 7, JCM 8608; 8, JCM 8677; 9, JCM 20024; 10, LMG 17315; 10 strains of *L. paracasei* (B) - M, DNA molecular marker; 1, LMG 13087; 2, LMG 9193; 3, LMG 9438; 4, LMG 11459; 5, LMG 11961; 6, LMG 12164; 7, LMG 19719; 8, JCM 1163; 9, LMG 9191; 10, JCM 20315; and 10 strains of *L. rhamnosus* (C) - M, DNA molecular marker; 1, LMG 6400; 2, LMG 8153; 3, LMG 10768; 4, LMG 10772; 5, LMG 12166; 6, LMG 18030; 7*,* LMG 23304; 8, LMG 23536; 9, LMG 23550; 10, LMG 25881.

**Figure S8**

AFLP fingerprint profiles generated for *Lactobacillus casei* strains: LMG 6904, LMG 23516, LMG 24099, LMG 24102, JCM 2120, JCM 8129, JCM 8608, JCM 8677, JCM 20024 and LMG 17315.

**Figure S9**

AFLP fingerprint profiles generated for *Lactobacillus paracasei* strains: LMG 13087, LMG 9193, LMG 9438, LMG 11459, LMG 11961, LMG 12164, LMG 19719, JCM 1163, LMG 9191 and JCM 20315.

**Figure S10**

AFLP fingerprint profiles generated for *Lactobacillus rhamnosus* strains: LMG 6400, LMG 8153, LMG 10768, LMG 10772, LMG 12166, LMG 18030*,* LMG 23304, LMG 23536, LMG 23550 and LMG 25881.
