# Supplementary material for: Molecular Routes to Specific Identification of the Lactobacillus Casei Group at the Species, Subspecies and Strain Level
Source: Int J Mol Sci. 2020 Apr 13;21(8):2694. doi: 10.3390/ijms21082694 (PMC7216162; doi:10.3390/ijms21082694)
Supplement: Supplementary file 1 [file ijms-21-02694-s001.zip › ijms-752512-Proofdone sup/Table S1.pdf]

Table S1

Detailed information about polymerase chain reactions conducted in this study.

Primers	Sequences	PCR conditions	Amplification mixture	Amplified region (predicted PCR product length)	Application	References
Genus-, species-, subspecies-specific PCR						
LbLMA1-rev<br>R16-1	5’-CTCAAAACTAAACAAAGTTTC-3’<br>5’-CTTGTACACACCGCCCGTCA-3’	ID[1]: 95°C – 5 min<br>D[2]: 95°C – 30 sec<br>A[3]: 55°C – 30 sec<br>E[4]: 72°C – 30 sec<br>FE[5]: 72°C – 7 min<br>Number of cycles - 30	0.5 µM each primer,<br>2x PCR Master Mix Plus[6] (Taq DNA polymerase - 0,1U/µl, $MgCl_2$ - 4mM, dNTPs - 0,5 mM), 50 ng DNA, reaction vol. 20 µl	16S-23S spacer region rDNA (250 bp)	Identification of the genus *Lactobacillus*	[1]
TUF-1<br>TUF-2	5’-GATGCTGCTCCAGAAGA-3’<br>5’-ACCTTCTGGCAATTCAATC-3’	ID: 95°C – 3 min<br>D: 95°C – 30sec<br>A: 52°C – 30 sec<br>E: 72°C – 2 min<br>FE: 72°C – 10 min<br>Number of cycles - 30	1 µM each primer,<br>2x PCR Master Mix Plus (Taq DNA polymerase - 0,1U/µl, $MgCl_2$ - 4mM, dNTPs - 0,5 mM), 50 ng DNA, reaction vol. 20 µl	*tuf* gene encoding elongation factor Tu<br>(800 bp)	Identification of the genus *Lactobacillus*	[2]
LCgprpoA-F2<br>LCgprpoA-R2	5’-CACTCAARATGAAYACYGATGA-3’<br>5’-CGTGGTGAGATTGAGCCAT-3’	ID: 94°C – 5 min<br>D: 94°C – 1 min<br>A: 61°C – 1 min<br>E: 72°C – 1.5min<br>FE: 72°C – 7 min<br>Number of cycles - 25	1 µM each primer,<br>2x PCR Master Mix Plus (Taq DNA polymerase - 0,1U/µl, $MgCl_2$ - 4mM, dNTPs - 0,5 mM), 50 ng DNA, reaction vol. 20 µl	*rpoA* gene<br>(364 bp)	Identification of bacteria belong to *Lactobacillus casei* group	[3]
Y2<br>casei (W1)<br>para (W2)<br>rham (W3)<br>zeae (D1)	5’-CCCACTGCTGCCTCCCGTAGGAGT-3’<br>5’-TGCACTGAGATTCGACTTAA-3’<br>5’-CACCGAGATTCAACATGG-3’<br>5’-TGCATCTTGATTTAATTTTG-3’<br>5’-TGCATCGTGATTCAACTTAA-3’	ID: 94°C – 3 min<br>D: 94°C – 45 sec<br>A: 60°C – 45 sec<br>E: 72°C – 1 min<br>FE: 72°C – 5 min<br>Number of cycles - 30	0.5 µM universal primer and 0.5 µM species-specific primer,<br>2x Dream Taq Green PCR Master Mix[7] (Dream Taq DNA polymerase - 0,1U/µl, $MgCl_2$ - 4mM, dNTPs - 0,4 mM), 50 ng DNA, reaction vol. 20 µl	16S rRNA gene<br>(290 bp)	Identification of bacteria belong to *L. casei, L. paracasei*, *L. rhamnosus* and *„L. zeae”*	[4,5]
SpeOPT16zeae-F<br>SpeOPT16zeae-R	5’-CCATCACGGCAGAAGAAAC-3’<br>5’-GCTTCCACCTGCTTATTGAC-3’	ID: 94°C – 5 min<br>D: 94°C – 1 min<br>A: 66°C – 1 min<br>E: 72°C – 1.5min<br>FE: 72°C – 7 min<br>Number of cycles - 25	1 µM each primer,<br>2x Dream Taq Green PCR Master Mix (Dream Taq DNA polymerase - 0,1U/µl, $MgCl_2$ - 4mM, dNTPs - 0,4 mM), 50 ng DNA, reaction vol. 20 µl	bacteriophage gene<br>(451 bp)	Identification of bacteria belong to *„L. zeae”*	[6]
SpeOPT14rha-F<br>SpeOPT14rha-R	5’-AGATGTTAGTTGCCGTACTGCC-3’<br>5’-GCCGTTGACTATGCCCTTG-3’	ID: 94°C – 5 min<br>D: 94°C – 1 min<br>A: 66°C – 1 min<br>E: 72°C – 1.5min	1 µM each primer,<br>2x Dream Taq Green PCR Master Mix (Dream Taq DNA polymerase - 0,1U/µl,	rhamnulokinase gene<br>(102 bp)	Identification of bacteria belong to *L. rhamnosus*	[6]

		FE: 72°C – 7 min<br>Number of cycles - 25	$MgCl_2$ - 4mM, dNTPs - 0,4 mM), 50 ng DNA, reaction vol. 20 µl			
SpeOPT11tol-F<br>SpeOPT11tol-R	5’-CTCCTACGACTTAAATTAACCTGCT-3’<br>5’-CAGGGGTGCTTTTCTTTGCATG-3’	ID: 94°C – 5 min<br>D: 94°C – 1 min<br>A: 66°C – 1 min<br>E: 72°C – 1.5min<br>FE: 72°C – 7 min<br>Number of cycles - 25	1 µM each primer,<br>2x Dream Taq Green PCR Master Mix (Dream Taq DNA polymerase - 0,1U/µl, $MgCl_2$ - 4mM, dNTPs - 0,4 mM), 50 ng DNA, reaction vol. 20 µl	Mobilisation protein gene (179 bp)	Identification of bacteria belong to *L. paracasei* subsp. *tolerans*	[6]
			Multiplex PCR			
CPR<br>CAS<br>PAR<br>RHA	5’-CAANTGGATNGAACCTGGCTTT-3’<br>5’-ACTGAAGGCGACAAGGA-3’<br>5’-GACGGTTAAGATTGGTGAC-3’<br>5’-GCGTCAGGTTGGTGTTG-3’	ID: 95°C – 5 min<br>D: 95°C – 30 sec<br>A: 54°C – 1 min<br>E: 72°C – 1.5min<br>FE: 72°C – 7 min<br>Number of cycles - 30	0.2 µM each primer<br>2x Dream Taq Green PCR Master Mix (Dream Taq DNA polymerase - 0,1U/µl, $MgCl_2$ - 4mM, dNTPs - 0,4 mM), 25 ng DNA, reaction vol. 20 µl	*tuf* gene<br>L. casei (700, 540, 350 bp),<br>L. paracasei (540, 240 bp)<br>L. rhamnosus (540 bp)	Identification of bacteria belong to *L. casei, L. paracasei* and *L. rhamnosus*	[2]
CZfor<br>PC2a<br>RHfor<br>CPRrev	5’-CAGCGCTGGTGGAAGACTTG-3’<br>5’-GGATTGGGTTTTGCGTGATGGTCGC-3’<br>5’-GACTTCTCAACCAGCAGCGCAGA-3’<br>5’-TGCATTTCCCCGCTTTCATGACT-3’	ID: 94°C – 5 min<br>D: 94°C – 30 sec<br>A: 68°C – 30 sec<br>E: 72°C – 45 sec<br>FE: 72°C – 5 min<br>Number of cycles - 35	0.25 µM each primer, Dream Taq DNA polymerase[8] - 1.25 U, dNTPs[9] - 0,2 mM, DreamTaq Buffer (10x): 20mM $MgCl_2$, 25 ng DNA, reaction vol. 20 µl	*mutL* gene,<br>*L. casei* (666 bp),<br>*L. paracasei* (253 bp),<br>L. *rhamnosus* (801 bp)	Identification of bacteria belong to *L. casei, L. paracasei* and *L. rhamnosus*	[7]
			HRM analysis			
poxcDNAFw<br>poxPromRv	5’-CAGACGCAATGATCAAGGTG-3’<br>5’-AATGCGCCYACTTCTTCATG-3’	ID: 98°C – 1min<br>D: 95°C – 15 sec<br>A/E: 60°C – 1 min<br>Number of cycles – 50<br>PCR product melting analysis:<br>95°C – 10 sec<br>60°C – 1 min<br>65°C - 95°C, 5 sec/step<br>Temperature increments of 0.2°C	0.5 µM each primer,<br>2x SsoFast EvaGreen Supermix[10] (2x reaction buffer with dNTPs, Sso7d-fusion polymerase, EvaGreen dye and stabilizers), 15 ng DNA, reaction vol. 15 µl	*spxB* gene (260 bp)	Identification of bacteria belong to *L. casei, L. paracasei* and *L. rhamnosus*	[8]
GroHRM-F<br>GroHRM-R	5’-GTTTGATCGCGGCTATCTGA-3’<br>5’-CCTTGTTGMACGATTTCTTG-3’	ID: 98°C – 1min<br>D: 95°C – 30 sek<br>A: 59°C – 20 sek<br>E: 72°C – 1 sek<br>Number of cycles – 45<br>PCR product melting analysis:<br>65°C - 95°C, 5 sec/step<br>Temperature increments of 0.2°C	0.25 µM each primer,<br>2x SsoFast EvaGreen Supermix (2x reaction buffer with dNTPs, Sso7d-fusion polymerase, EvaGreen dye and stabilizers), 15 ng DNA, reaction vol. 15 µl	*groEL* gene (150 bp)	Identification of bacteria belong to *L. casei, L. paracasei* and *L. rhamnosus*	[9]

RAPD-PCR						
80A_RAPD	5'-AGTCAGCCAC-3'	ID: 94°C – 5 min<br>D: 94°C – 1 min<br>A: 32°C – 2 min<br>E: 72°C – 2 min<br>FE: 72°C – 5 min<br>Number of cycles - 35	0.4 µM of the primer, Dream Taq DNA polymerase - 1 U, dNTPs - 0,2 mM, DreamTaq Buffer (10x): 20mM $MgCl_2$, 100 ng DNA, reaction vol. 40 µl	Random amplification of polymorphic DNA fragments	Differentiation of bacteria belong to L. casei group at strain level	[10]
80B_RAPD_M13	5'-GAGGGTGGCGGTTCT-3'	ID: 94°C – 2 min<br>D: 94°C – 1 min<br>A: 42°C – 20 sec<br>E: 72°C – 2 min<br>FE: 72°C – 10 min<br>Number of cycles - 40	0.4 µM of the primer, Dream Taq DNA polymerase – 1.25 U, dNTPs - 0,2 mM, DreamTaq Buffer (10x): 20mM $MgCl_2$, 100 ng DNA, reaction vol. 40 µl	Random amplification of polymorphic DNA fragments	Differentiation of bacteria belong to L. casei group at strain level	[11]
80C_RAPD_OPT-14	5'-AATGCCGCAG-3'	ID: 94°C – 5 min<br>D: 94°C – 1 min<br>A: 36°C – 1 min<br>E: 72°C – 2 min<br>FE: 72°C – 7 min<br>Number of cycles - 45	0.4 µM of the primer, Dream Taq DNA polymerase – 1 U, dNTPs - 0,2 mM, DreamTaq Buffer (10x): 20mM $MgCl_2$, 100 ng DNA, reaction vol. 40 µl	Random amplification of polymorphic DNA fragments	Differentiation of bacteria belong to L. casei group at strain level	[6]
80D_RAPD_OPA-18	5'-AGGTGACCGT-3'	ID: 94°C – 5 min<br>D: 94°C – 15 sec<br>A: 40°C – 30 sec<br>E: 72°C – 1 min<br>FE: 72°C – 8 min<br>Number of cycles - 30	0.4 µM of the primer, Dream Taq DNA polymerase – 1 U, dNTPs - 0,2 mM, DreamTaq Buffer (10x): 20mM $MgCl_2$, 100 ng DNA, reaction vol. 40 µl	Random amplification of polymorphic DNA fragments	Differentiation of bacteria belong to L. casei group at strain level	[12–14]
Rep-PCR						
BOXA1R	5'-CTACGGCAAGGCGACGCTGACG-3'	ID: 94°C – 5 min<br>D: 94°C – 30 sec<br>A: 50°C – 1 min<br>E: 72°C – 4 min<br>FE: 72°C – 7 min<br>Number of cycles - 35	1 µM of the primer, Dream Taq DNA polymerase – 2.5 U, dNTPs - 0,2 mM, DreamTaq Buffer (10x): 20mM $MgCl_2$, 100 ng DNA, reaction vol. 50 µl	Amplification of repetitive BOX elements	Differentiation of bacteria belong to L. casei group at strain level	[15,16]
$(GTG)_5$	5'-GTGGTGGTGGTGGTG-3'	ID: 94°C – 5 min<br>D: 94°C – 30 sec<br>A: 45°C – 1 min<br>E: 72°C – 4 min<br>FE: 72°C – 7 min<br>Number of cycles - 35	1 µM of the primer, Dream Taq DNA polymerase – 2.5 U, dNTPs - 0,2 mM, DreamTaq Buffer (10x): 20mM $MgCl_2$, 100 ng DNA, reaction vol. 50 µl	Amplification of polytrinucleotides – $(GTG)_5$	Differentiation of bacteria belong to L. casei group at strain level	[15,16]
ERIC I<br>ERIC II	5'-ATGTAAGCTCCTGGGGATTCAC-3'<br>5'-AAGTAAGTGACTGGGGTGAGCG-3'	ID: 94°C – 3 min<br>D: 94°C – 30 sec<br>A: 48°C – 1 min<br>E: 72°C – 5 min<br>FE: 72°C – 7 min<br>Number of cycles - 35	1 µM each primer, Dream Taq DNA polymerase – 5 U, dNTPs - 0,2 mM, DreamTaq Buffer (10x): 20mM $MgCl_2$, 100 ng DNA, reaction vol. 50 µl	Amplification of repetitive ERIC sequences	Differentiation of bacteria belong to L. casei group at strain level	[15,16]

RFLP-PCR						
Primers	Sequences	PCR conditions	Amplification mixture	PCR product length/ digestion mixture	Digestion conditions	References
F1<br>R1530	5’-AGAGTTTGATCCTGGCTCAG-3’<br>5’-AAGGAGGTGATCCAGCCGCA-3’	ID: 94°C – 3 min<br>D: 94°C – 1 min<br>A: 58°C – 1min<br>E: 72°C – 1.5 min<br>FE: 72°C – 10 min<br>Number of cycles - 35	1 µM each primer,<br>2x Dream Taq PCR Master Mix[11] (Dream Taq DNA polymerase - 0,1U/µl, $MgCl_2$ - 4mM, dNTPs - 0,4 mM), 62.5 ng DNA, reaction vol. 50 µl	16S rRNA gene (1560 bp), RFLP reaction mixture: PCR product – 20 µl, 10X Buffer R - 4 µl, MseI[12] (10 U), reaction vol. 60 µl	Incubation at 65°C for 16h, inactivation by adding 0,5 M EDTA, A, pH 8.0 to achieve a 20 mM final concentration	[17,18]
TUF-1<br>TUF-2	5’-GATGCTGCTCCAGAAGA-3’<br>5’-ACCTTCTGGCAATTCAATC-3’	ID: 94°C – 3 min<br>D: 94°C – 1 min<br>A: 52°C – 1 min<br>E: 72°C – 1.5 min<br>FE: 72°C – 10 min<br>Number of cycles - 35	1 µM each primer,<br>2x Dream Taq PCR Master Mix (Dream Taq DNA polymerase - 0,1U/µl, $MgCl_2$ - 4mM, dNTPs - 0,4 mM), 125 ng DNA, reaction vol. 50 µl	*tuf* gene (800 bp), RFLP reaction mixture: PCR product – 20 µl, 10X Buffer R - 4 µl, HaeIII[13] (10 U), reaction vol. 60 µl	Incubation at 37°C for 12h, inactivation by incubation at 80°C for 20min	[2,19]
LCdnak-55F<br>LCdnak-1050R	5’-GCCATTGGMCGTTCMCCTTG-3’<br>5’-CARGAAATTTCWGCGATGAT-3’	ID: 94°C – 3 min<br>D: 94°C – 1 min<br>A: 55°C – 1min<br>E: 72°C – 1.5 min<br>FE: 72°C – 10 min<br>Number of cycles - 35	1 µM each primer,<br>2x Dream Taq PCR Master Mix (Dream Taq DNA polymerase - 0,1U/µl, $MgCl_2$ - 4mM, dNTPs - 0,4 mM), 62.5 ng DNA, reaction vol. 50 µl	*dnaK* gene (995 bp), RFLP reaction mixture: PCR product – 20 µl, 10X Buffer Tango - 4 µl, ApoI[14] (10 U), reaction vol. 60 µl	Incubation at 37°C for 16h, inactivation by incubation at 80°C for 20min	[20]

[1] Initial denaturation, [2] denaturation, [3] annealing, [4] extension, [5] final elongation,

[6] 2xPCR Master Mix Plus (A&A Biotechnology, catalog number: 2005-100P),

[7] 2xDream Taq Green PCR Master Mix (Thermo Scientific, catalog number: K1081),

[8] DreamTaq DNA Polymerase 5 U/µl (Thermo Scientific, catalog numer: EP0702),

[9] dNTP Mix, 10 mM each (Thermo Scientific, catalog numer: R0191),

[10] SsoFast EvaGreen Supermix (Biorad, catalog numer: 172-5200),

[11] 2xDream Taq PCR Master Mix (Thermo Scientific, catalog number: K1072),

[12] MseI 10U/µl (Thermo Scientific, catalog number: ER0981),

[13] HaeIII 10U/µl (Thermo Scientific, catalog number: ER0151),

[14] ApoI 10U/µl (Thermo Scientific, catalog number: ER1381).

References

1. Dubernet, S.; Desmasures, N.; Guéguen, M. A PCR-based method for identification of lactobacilli at the genus level. *FEMS Microbiol. Lett.* **2002**, *214*, 271–275.
2. Ventura, M.; Canchaya, C.; Meylan, V.; Klaenhammer, T.R.; Zink, R. Analysis, Characterization, and loci of the tuf genes in *Lactobacillus* and *Bifidobacterium* species and their direct application for species Identification. *Appl. Environ. Microbiol.* **2003**, *69*, 6908–6922.
3. Huang, C.H.; Chang, M.T.; Huang, M.C.; Lee, F.L. Application of the SNaPshot minisequencing assay to species identification in the *Lactobacillus case*i group. *Mol. Cell. Probes* **2011**, *25*, 153–157.
4. Ward, L.J.H.; Timmins, M.J. Differentiation of *Lactobacillus casei, Lactobacillus paracasei* and *Lactobacillus rhamnosus* by polymerase chain reaction. *Lett. Appl. Microbiol.* **1999**, *29*, 90–92.
5. Desai, A.R.; Shah, N.P.; Powell, I.B. Discrimination of dairy industry isolates of the *Lactobacillus casei* group. *J. Dairy Sci.* **2006**, *89*, 3345–3351.
6. Huang, C.-H.; Lee, F.-L. Development of novel species-specific primers for species identification of the *Lactobacillus casei* group based on RAPD fingerprints. *J. Sci. Food Agric.* **2009**, *89*, 1831–1837.
7. Bottari, B.; Felis, G.E.; Salvetti, E.; Castioni, A.; Campedelli, I.; Torriani, S.; Bernini, V.; Gatti, M. Effective identification of *Lactobacillus casei* group species: Genome-based selection of the gene mutL as the target of a novel multiplex PCR assay. *Microbiol. (United Kingdom)* **2017**, *163*, 950–960.
8. Savo Sardaro, M.L.; Levante, A.; Bernini, V.; Gatti, M.; Neviani, E.; Lazzi, C. The *spxB* gene as a target to identify *Lactobacillus casei* group species in cheese. *Food Microbiol.* **2016**, *59*, 57–65.
9. Koirala, R.; Taverniti, V.; Balzaretti, S.; Ricci, G.; Fortina, M.G.; Guglielmetti, S. Melting curve analysis of a *groEL* PCR fragment for the rapid genotyping of strains belonging to the *Lactobacillus casei* group of species. *Microbiol. Res.* **2015**, *173*, 50–58.
10. Tynkkynen, S.; Satokari, R.; Saarela, M.; Mattila-Sandholm, T.; Saxelin, M. Comparison of ribotyping, randomly amplified polymorphic DNA analysis, and pulsed-field gel electrophoresis in typing of *Lactobacillus rhamnosus* and *L. casei* strains. *Appl. Environ. Microbiol.* **1999**, *65*, 3908–14.
11. Rossetti, L.; Giraffa, G. Rapid identification of dairy lactic acid bacteria by M13-generated, RAPD-PCR fingerprint databases. *J. Microbiol. Methods* **2005**, *63*, 135–144.
12. Roy, D.; Ward, P.; Vincent, D.; Mondou, F. Molecular identification of potentially probiotic lactobacilli. *Curr. Microbiol.* **2000**, *40*, 40–46.
13. Vincent, D.; Roy, D.; Mondou, F.; Déry, C. Characterization of bifidobacteria by random DNA amplification. *Int. J. Food Microbiol.* **1998**, *43*, 185–193.
14. Daud Khaled, A.K.; Neilan, B.A.; Henriksson, A.; Conway, P.L. Identification and phylogenetic analysis of *Lactobacillus* using multiplex RAPD-PCR. *FEMS Microbiol. Lett.* **1997**, *153*, 191–

7.

15. Versalovic, J.; de Bruijn, F.J.; Lupski, J.R. Repetitive Sequence-based PCR (rep-PCR) DNA Fingerprinting of Bacterial Genomes. In *Bacterial Genomes*; Springer US, 1998; pp. 437–454.

16. Versalovic, J; Schneider, M.; deBruijn, F.J.; Lupski, J.R. Genomic fingerprinting of bacteria using repetitive sequence based polymerase chain reaction. *Methods Mol. Cell Biol.* **1994**, *5*, 25–40.

17. Dušková, M.; Šedo, O.; Kšicová, K.; Zdráhal, Z.; Karpíšková, R. Identification of lactobacilli isolated from food by genotypic methods and MALDI-TOF MS. *Int. J. Food Microbiol.* **2012**, *159*, 107–114.

18. Dec, M.; Puchalski, A.; Urban-Chmiel, R.; Wernicki, A. 16S-ARDRA and MALDI-TOF mass spectrometry as tools for identification of *Lactobacillus* bacteria isolated from poultry. *BMC Microbiol.* **2016**, *16*.

19. Park, S.H.; Jung, J.H.; Seo, D.H.; Lee, H.L.; Kim, G.W.; Park, S.Y.; Shin, W.C.; Hong, S.; Park, C.S. Differentiation of lactic acid bacteria based on RFLP analysis of the *tuf* gene. *Food Sci. Biotechnol.* **2012**, *21*, 911–915.

20. Huang, C.H.; Lee, F.L. The *dnaK* gene as a molecular marker for the classification and discrimination of the *Lactobacillus case*i group. *Antonie van Leeuwenhoek, Int. J. Gen. Mol. Microbiol.* **2011**, *99*, 319–327.
