# Supplementary figures and images for: Molecular Routes to Specific Identification of the Lactobacillus Casei Group at the Species, Subspecies and Strain Level
Source: Int J Mol Sci. 2020 Apr 13;21(8):2694. doi: 10.3390/ijms21082694 (PMC7216162; doi:10.3390/ijms21082694)

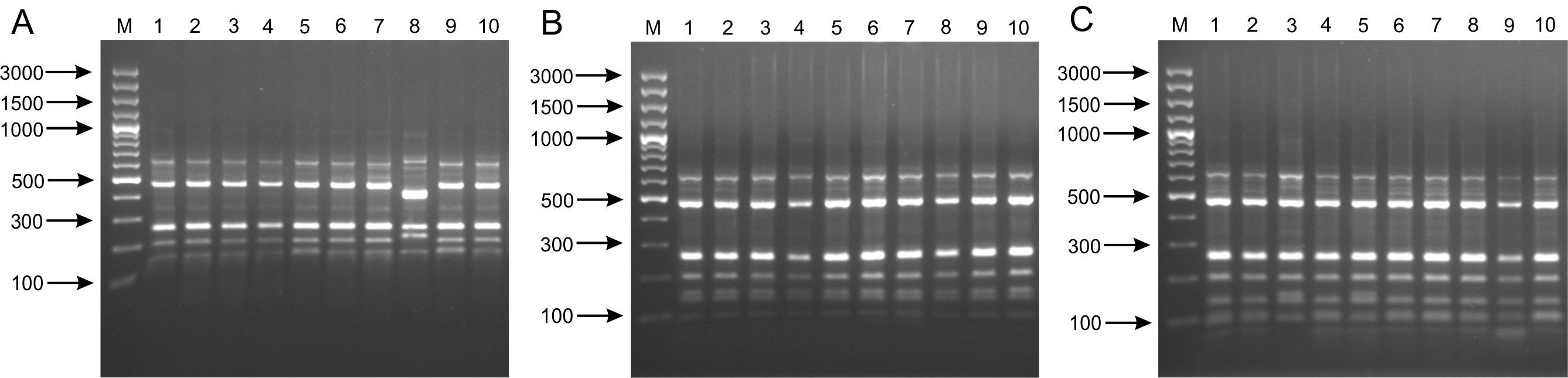

Supplement: Supplementary file 1 [file ijms-21-02694-s001.zip › ijms-752512-Proofdone sup/Figure S1.tif]

LMG 6400.fsa

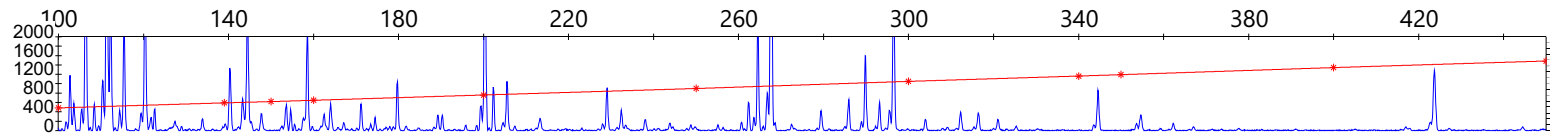

LMG 8153.fsa

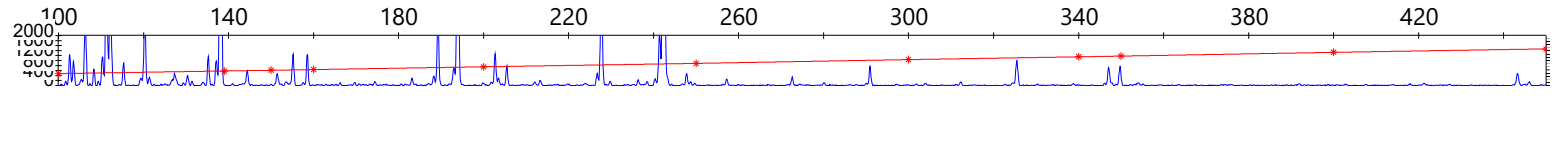

LMG 10768.fsa

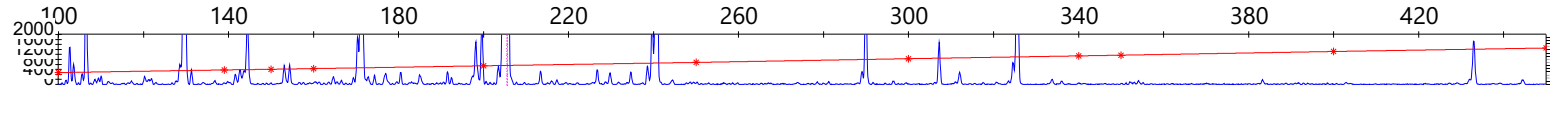

LMG 10772.fsa

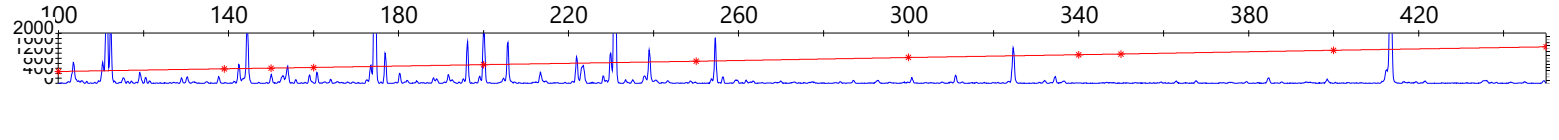

LMG 12166.fsa

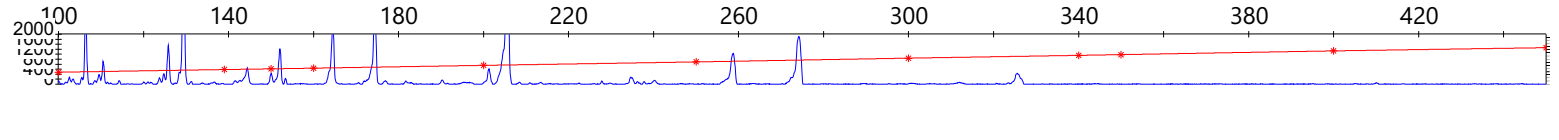

LMG 18030.fsa

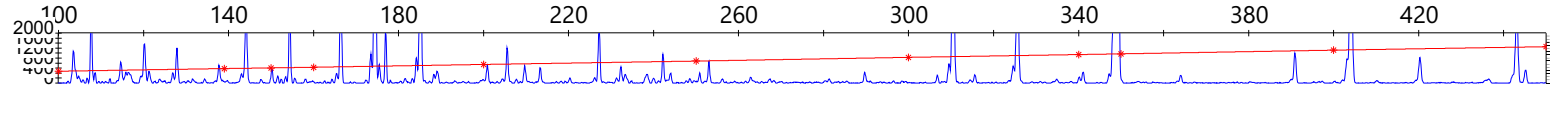

LMG 23304.fsa

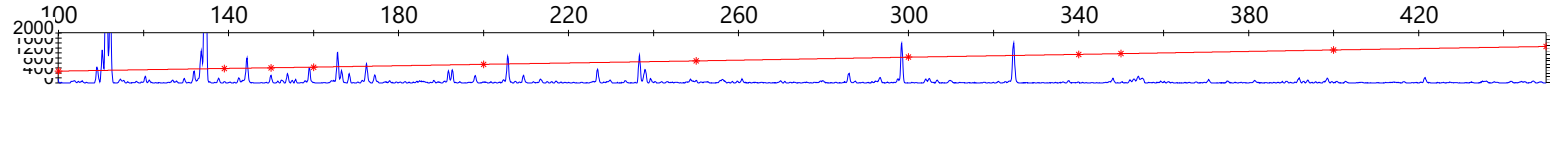

LMG 23536.fsa

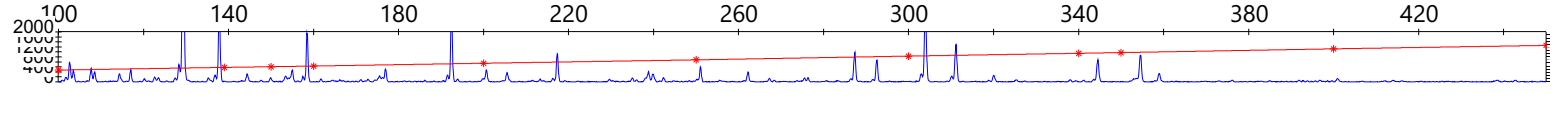

LMG 23550.fsa

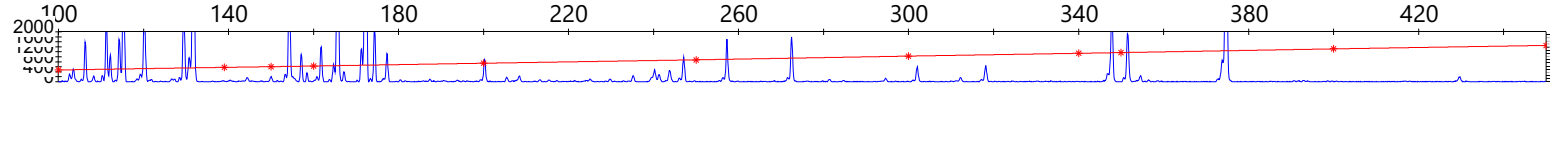

LMG 25881.fsa

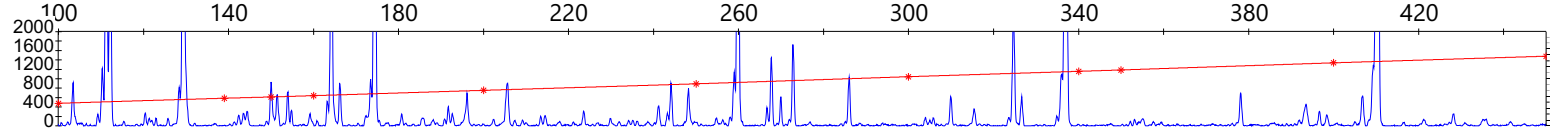

Supplement: Supplementary file 1 [file ijms-21-02694-s001.zip › ijms-752512-Proofdone sup/Figure S10.pdf]

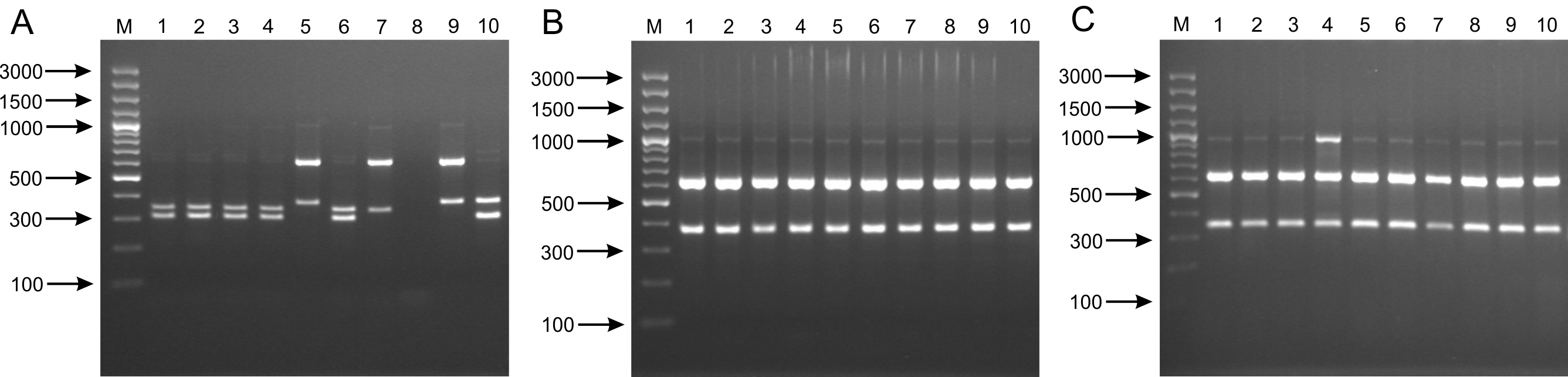

Supplement: Supplementary file 1 [file ijms-21-02694-s001.zip › ijms-752512-Proofdone sup/Figure S2.tif]

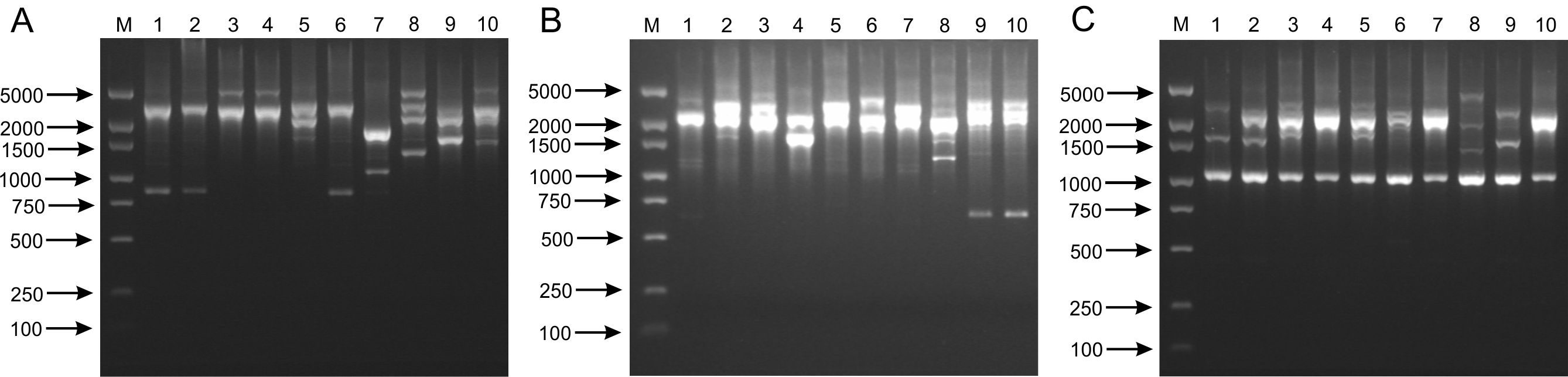

Supplement: Supplementary file 1 [file ijms-21-02694-s001.zip › ijms-752512-Proofdone sup/Figure S3.tif]

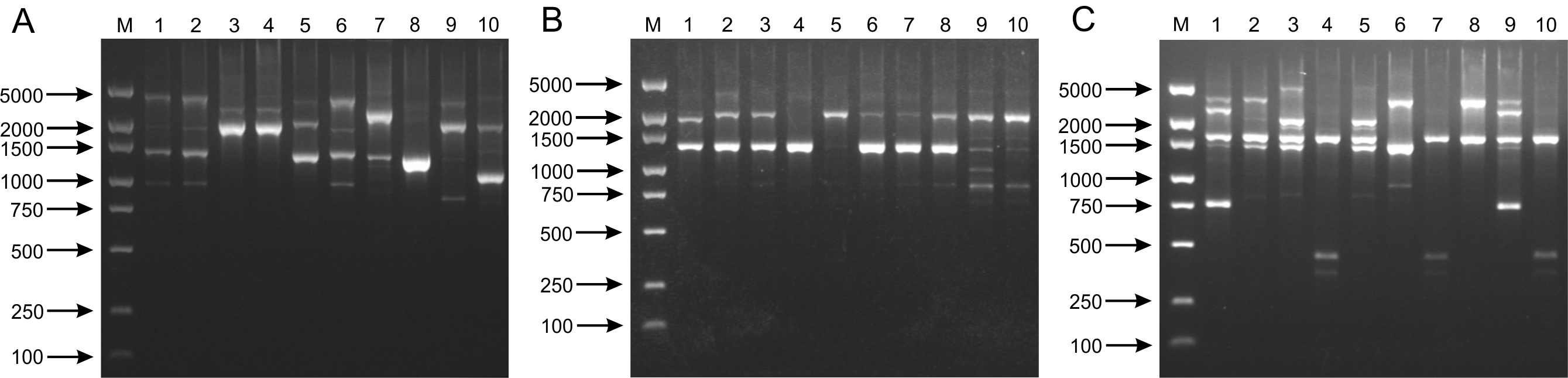

Supplement: Supplementary file 1 [file ijms-21-02694-s001.zip › ijms-752512-Proofdone sup/Figure S4.tif]

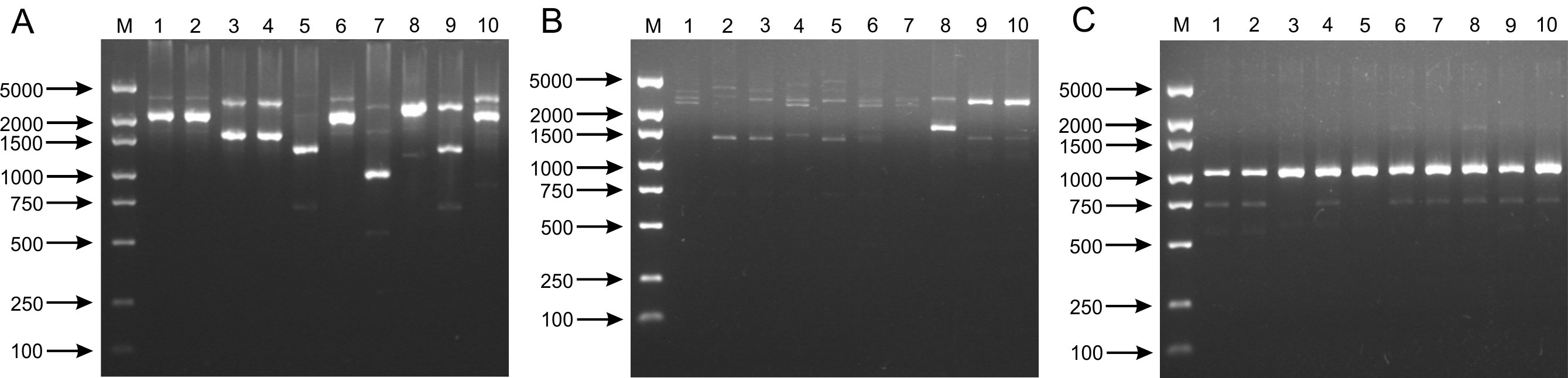

Supplement: Supplementary file 1 [file ijms-21-02694-s001.zip › ijms-752512-Proofdone sup/Figure S5.tif]

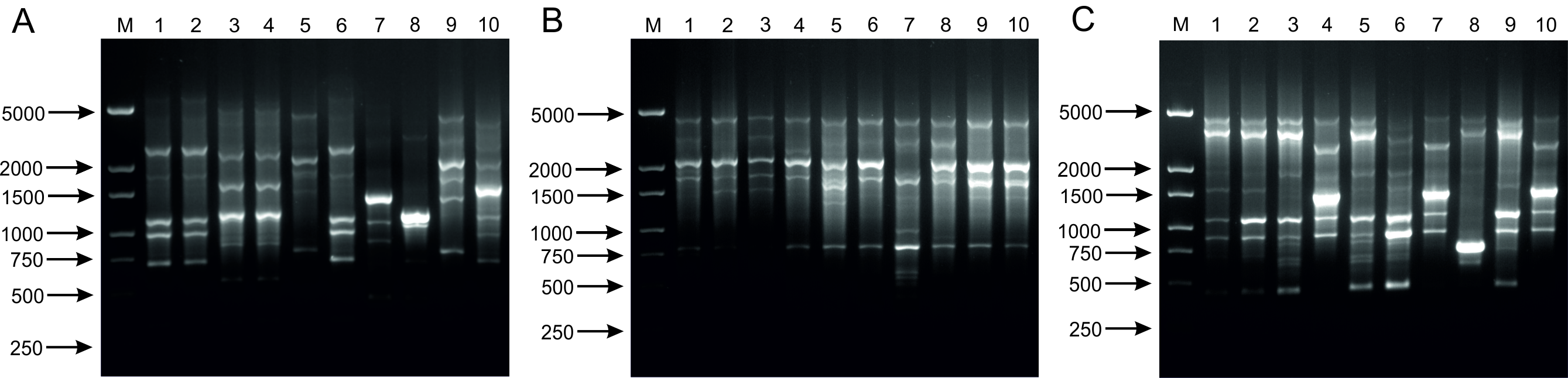

Supplement: Supplementary file 1 [file ijms-21-02694-s001.zip › ijms-752512-Proofdone sup/Figure S6.tif]

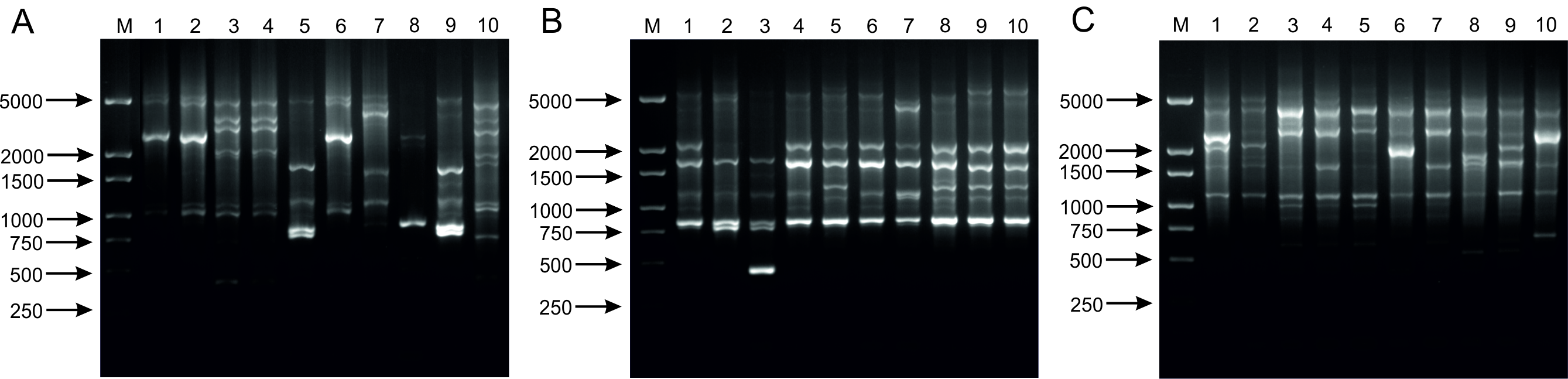

Supplement: Supplementary file 1 [file ijms-21-02694-s001.zip › ijms-752512-Proofdone sup/Figure S7.tif]

LMG 6904.fsa

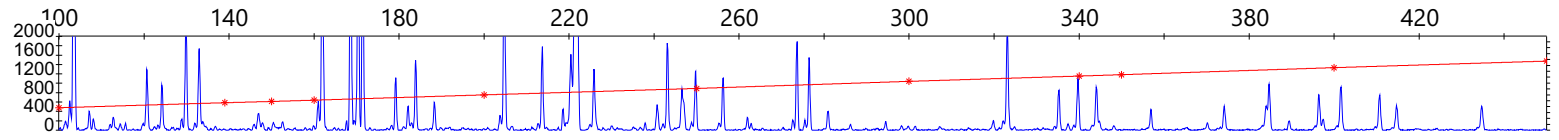

LMG 23516.fsa

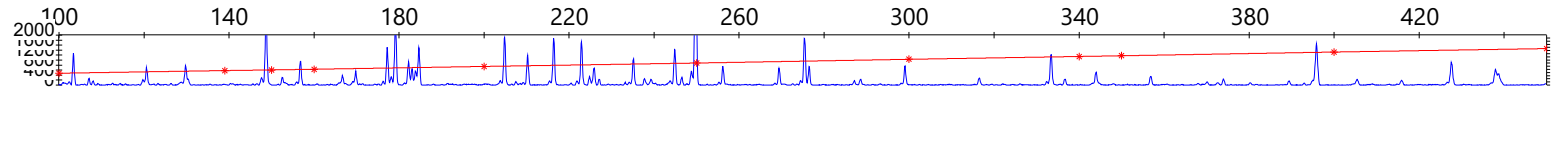

LMG 24099.fsa

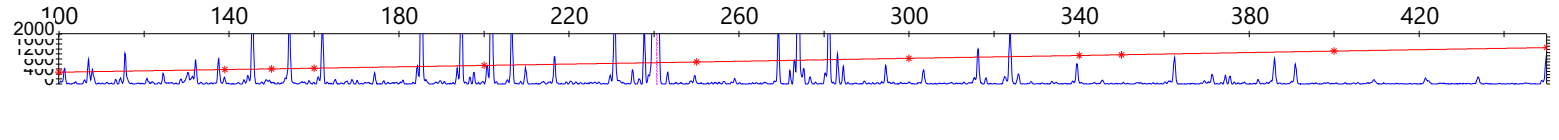

LMG 24102.fsa

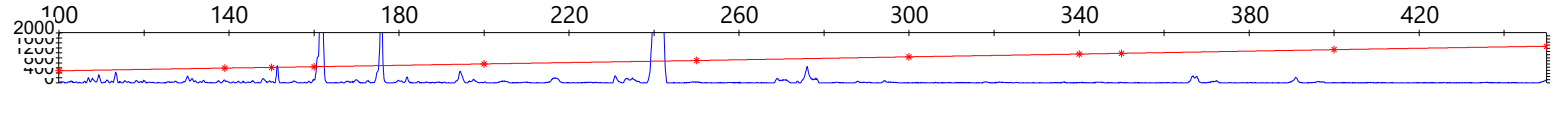

JCM 2120.fsa

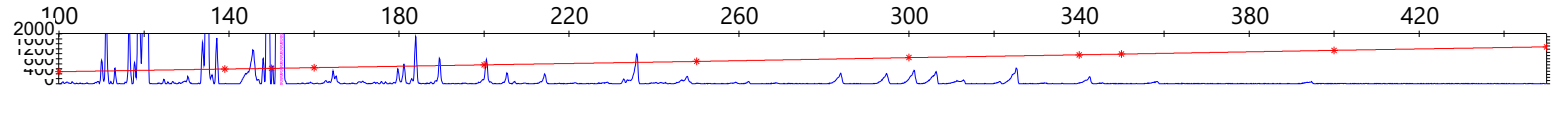

JCM 8129.fsa

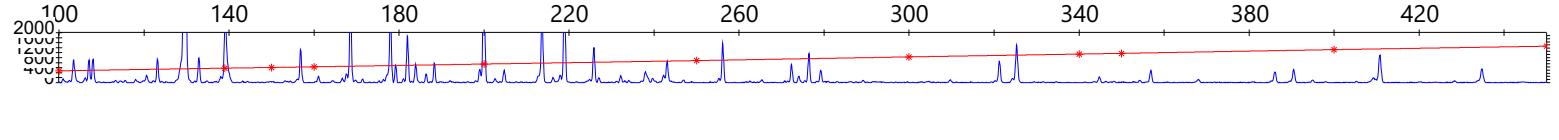

JCM 8608.fsa

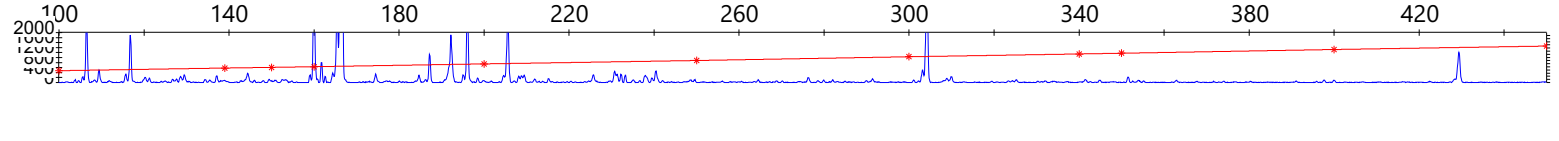

JCM 8677.fsa

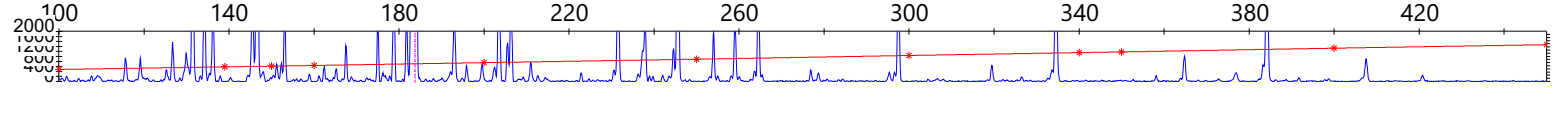

JCM 20024.fsa

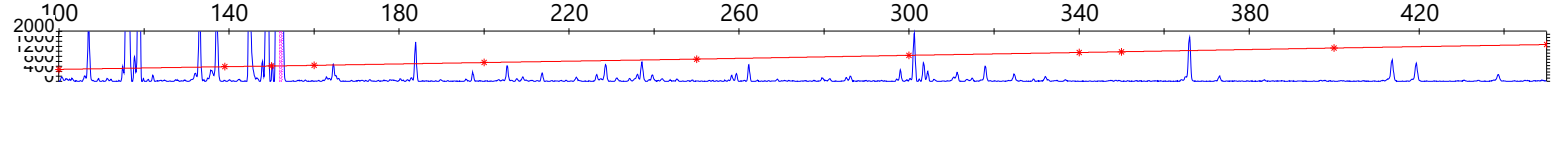

LMG 17315.fsa

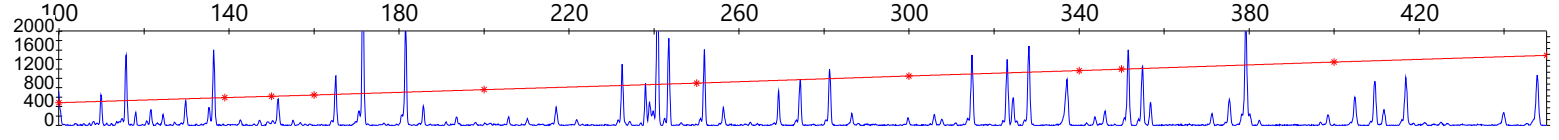

Supplement: Supplementary file 1 [file ijms-21-02694-s001.zip › ijms-752512-Proofdone sup/Figure S8.pdf]
