# Supplementary material for: Molecular Routes to Specific Identification of the Lactobacillus Casei Group at the Species, Subspecies and Strain Level
Source: Int J Mol Sci. 2020 Apr 13;21(8):2694. doi: 10.3390/ijms21082694 (PMC7216162; doi:10.3390/ijms21082694)

LMG 13087.fsa

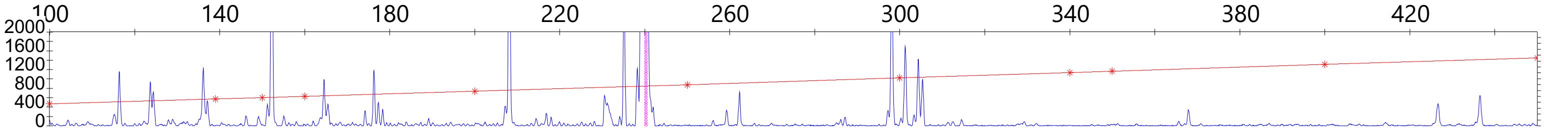

LMG 9193.fsa

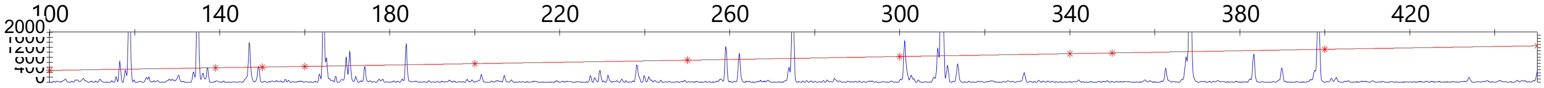

LMG 9438.fsa

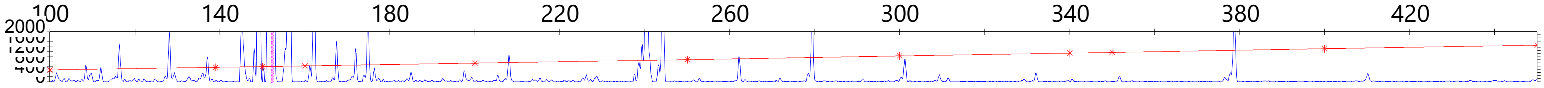

LMG 11459.fsa

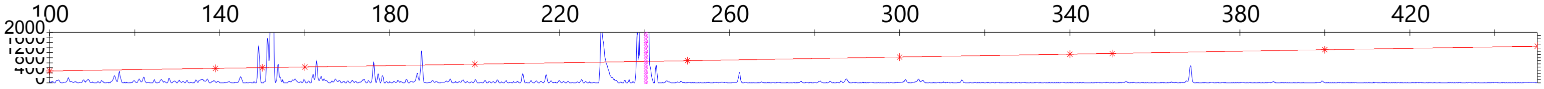

LMG 11961.fsa

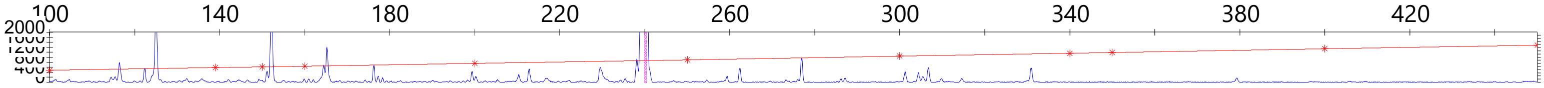

LMG 12164.fsa

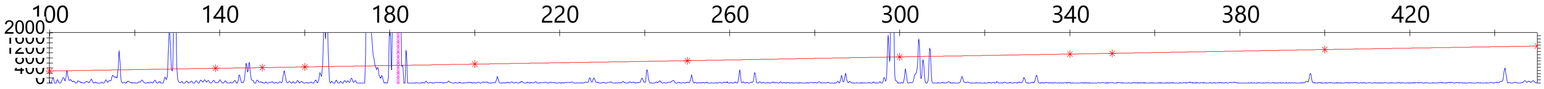

LMG 19719.fsa

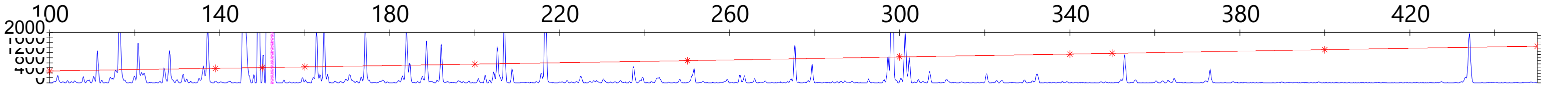

JCM 1163.fsa

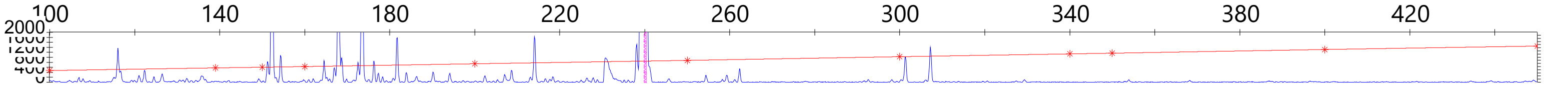

LMG 9191.fsa

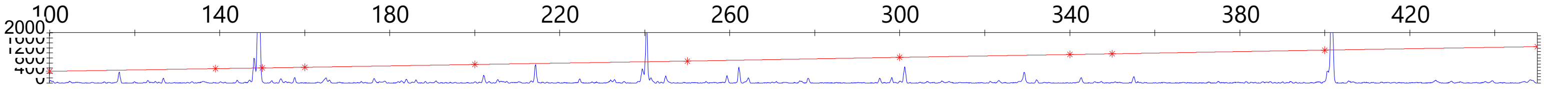

JCM 20315.fsa

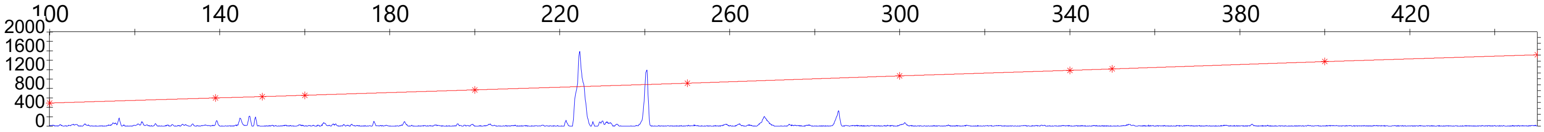

Supplement: Supplementary file 1 [file Figure S9.pdf › ijms-752512-Proofdone sup/Figure S9.pdf]
